# Supplementary material for: Chemical Constituents of Macaranga occidentalis, Antimicrobial and Chemophenetic Studies
Source: Molecules. 2022 Dec 12;27(24):8820. doi: 10.3390/molecules27248820 (PMC9782370; doi:10.3390/molecules27248820)
Supplement: Supplementary file 1 [file molecules-27-08820-s001.zip › molecules-2061640-supplementary.pdf]

SUPPLEMENTARY MATERIAL

Table of contents

Figure S1: ESI-HR Mass spectrum of **6a**

Figure S2: IR spectrum of **6a**

Figure S3: ^{1}H-NMR (500 MHz, acetone –*d*$_6$) spectrum of **6a**

Figure S4: ^{13}C-NMR (125 MHz, acetone –*d*$_6$) spectrum of **6a**

Figure S5: DEPT 135 spectrum of **6a**

Figure S6: ^{1}H-^{1}H COSY spectrum of **6a**

Figure S7: HSQC spectrum of **6a**

Figure S8: HMBC spectrum of **6a**

Figure S9: ESI-HR Mass spectrum of **6b**

Figure S10: ^{1}H-NMR (500 MHz, acetone –*d*$_6$) spectrum of **6b**

Figure S11: ^{13}C-NMR (125 MHz, acetone –*d*$_6$) spectrum of **6b**

Figure S12: DEPT 135 spectrum of **6b**

Figure S13: ^{1}H-^{1}H COSY spectrum of **6b**

Figure S14: HSQC spectrum of **6b**

Figure S15: HMBC spectrum of **6b**

Figure S16: ESI-HR Mass spectrum of **6c**

Figure S17: ^{1}H-NMR (500 MHz, acetone –*d*$_6$) spectrum of **6c**

Figure S18: ^{13}C-NMR (125 MHz, acetone –*d*$_6$) spectrum of **6c**

Figure S19: DEPT 135 spectrum of **6c**

Figure S20: ^{1}H-^{1}H COSY spectrum of **6c**

Figure S21: HSQC spectrum of **6c**

Figure S22: HMBC spectrum of **6c**

Figure S23: ^{1}H-NMR (500 MHz, $CDCl_3$) spectrum of **1+2**

Figure S24: ^{13}C-NMR (125 MHz, $CDCl_3$) spectrum of **1+2**

Figure S25: ^{1}H-NMR (500 MHz, Pyridine–*d*$_5$) spectrum of **3**

Figure S26: ^{13}C-NMR (125 MHz, Pyridine–*d*$_5$) spectrum of **3**

Figure S27: ^{1}H-NMR (500 MHz, $CDCl_3$) spectrum of **4**

Figure S28: ^{13}C-NMR (125 MHz, $CDCl_3$) spectrum of **4**

Figure S29: ^{1}H-NMR (500 MHz, acetone –*d*$_6$) spectrum of **5**

Figure S30: ^{13}C-NMR (125 MHz, acetone –*d*$_6$) spectrum of **5**

Figure S31: ^{1}H-NMR (500 MHz, acetone –*d*$_6$) spectrum of **6**

Figure S32: ^{13}C-NMR (1254 MHz, acetone –*d*$_6$) spectrum of **6**

Figure S33: ^{1}H-NMR (500 MHz, acetone –*d*$_6$) spectrum of **7**

Figure S34: ^{13}C-NMR (1254 MHz, acetone –*d*$_6$) spectrum of **7**

Figure S35: ^{1}H-NMR (500 MHz, acetone –*d*$_6$) spectrum of **8**

Figure S36: ^{13}C-NMR (1254 MHz, acetone –*d*$_6$) spectrum of **8**

Figure S37: ^{1}H-NMR (500 MHz, acetone –*d*$_6$) spectrum of **9**

Figure S38: ^{13}C-NMR (1254 MHz, acetone –*d*$_6$) spectrum of **9**

Figure S39: ^{1}H-NMR (500 MHz, acetone –*d*$_6$) spectrum of **10**

Figure S40: ^{13}C-NMR (1254 MHz, acetone –*d*$_6$) spectrum of **10**

Figure S41: NOESY spectrum of **10**

Figure S42: ^{1}H-NMR (500 MHz, Pyridine–*d*$_5$) spectrum of **11**

Figure S43: ^{13}C-NMR (125 MHz, Pyridine–*d*$_5$) spectrum of **11**

Figure S44: ^{1}H-NMR (500 MHz, DMSO–*d*$_6$) spectrum of **12**

Figure S45: ^{13}C-NMR (125 MHz, DMSO–*d*$_6$) spectrum of **12**

Figure S46: ^{1}H-NMR (500 MHz, Pyridine–*d*$_5$) spectrum of **13**

Figure S47: ^{13}C-NMR (125 MHz, Pyridine–*d*$_5$) spectrum of **13**

Figure S48: ^{1}H-NMR (500 MHz, Pyridine–*d*$_5$) spectrum of **14**

Figure S49: ^{13}C-NMR (125 MHz, Pyridine–*d*$_5$) spectrum of **14**

Figure S50: ^{1}H-NMR (500 MHz, CD_3OD) spectrum of **15**

Figure S51: ^{13}C-NMR (125 MHz, CD_3OD) spectrum of **15**

Figure S52: NOESY spectrum of **15**

Figure S53: ^{1}H-NMR (500 MHz, DMSO–*d*$_6$) spectrum of **16**

Figure S54: ^{13}C-NMR (125 MHz, *DMSO–d*$_6$) spectrum of **16**

Figure S55: NOESY spectrum of **16**

Figure S56: ^{1}H-NMR (500 MHz, $CDCl_3$-CD_3OD) spectrum of **17**

Figure S57: ^{13}C-NMR (125 MHz, $CDCl_3$-CD_3OD) spectrum of **17**

Scheme S1: Protocol of extraction and isolation of compounds from *M. occidentalis*

Measured Ion Mass(es) : 565.2560 **Deviation [mmu] :** 0.06 [mmu]

Calculated Ion Mass(es) : 565.25606 **Deviation [ppm] :** 0.11 [ppm]

Potential Molecular Formula : C34H38O6Na+

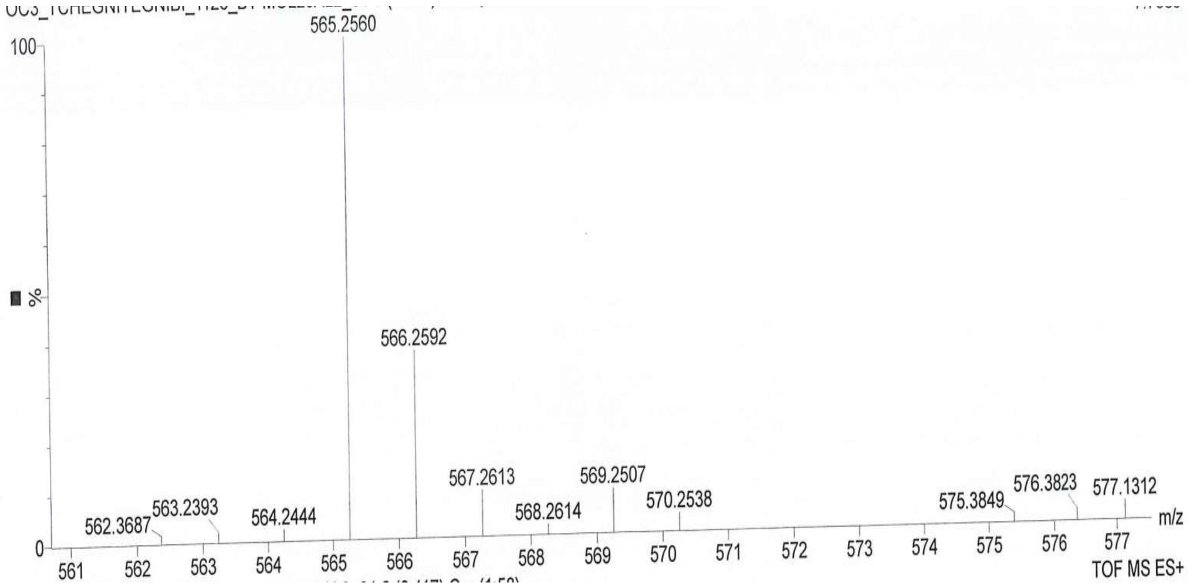

Figure S1: ESI-HR Mass spectrum of **6a**

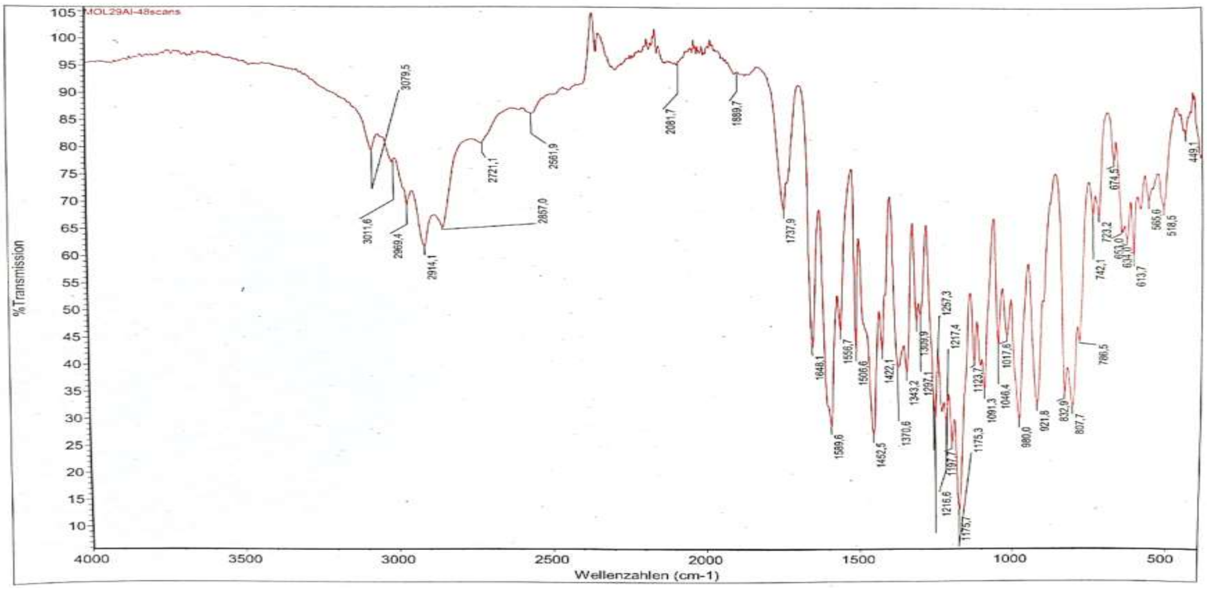

Figure S2: IR spectrum of **6a**

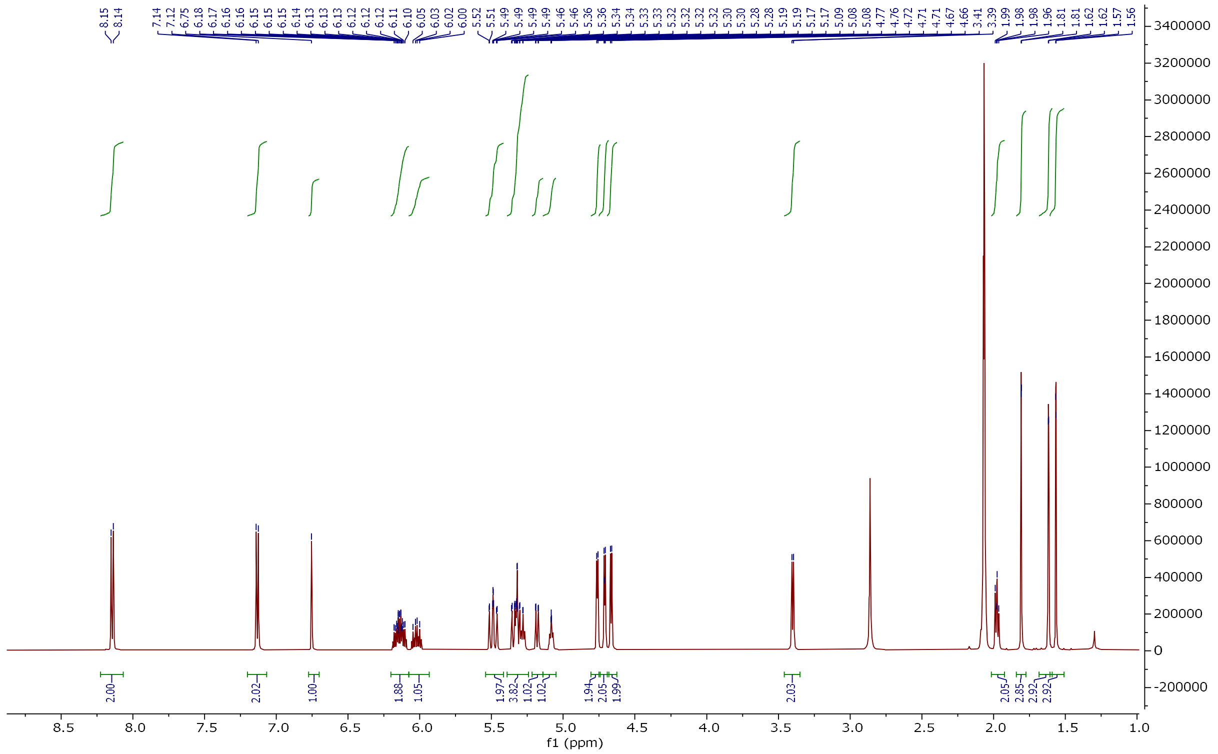

Figure S3: ^{1}H-NMR (500 MHz, acetone –d_6) spectrum of **6a**

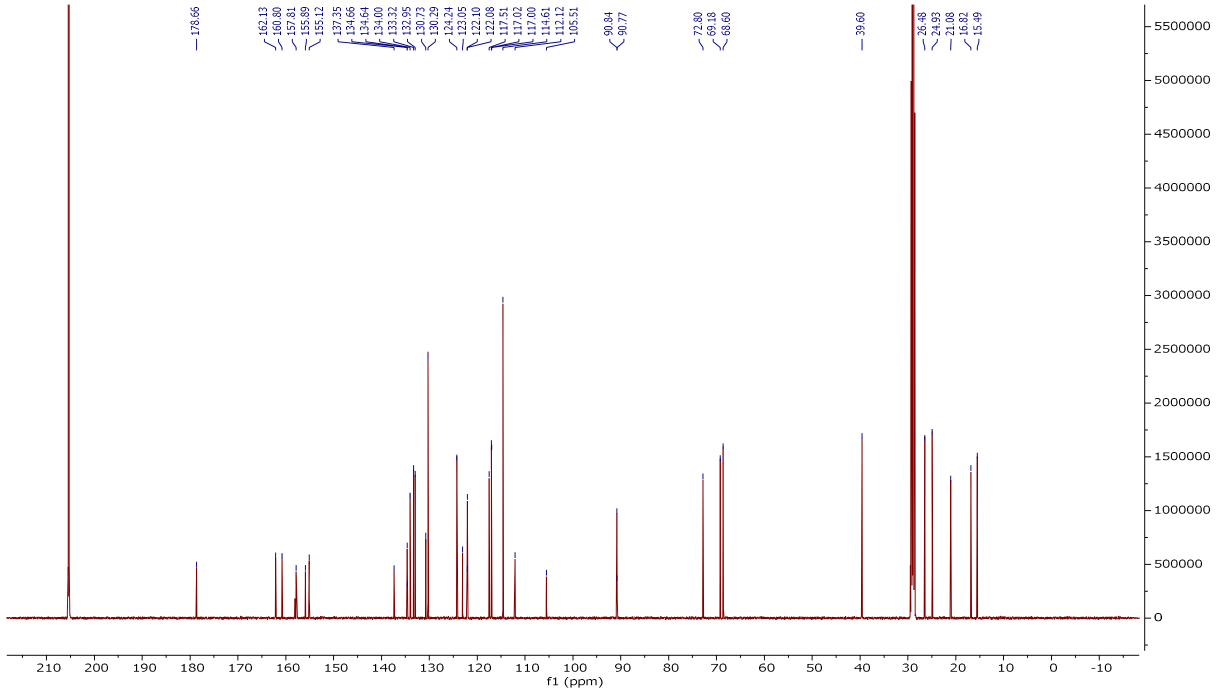

Figure S4: ^{13}C-NMR (125 MHz, acetone –d_6) spectrum of **6a**

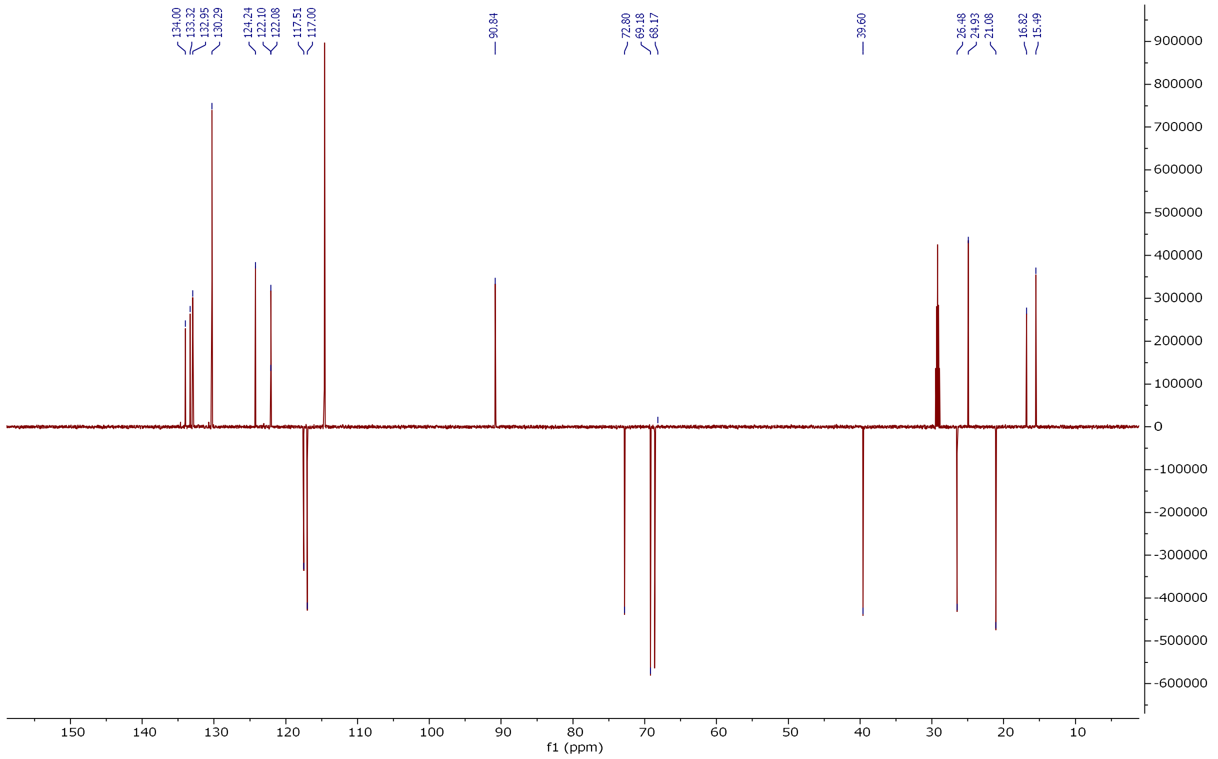

Figure S5: DEPT 135 spectrum of **6a**

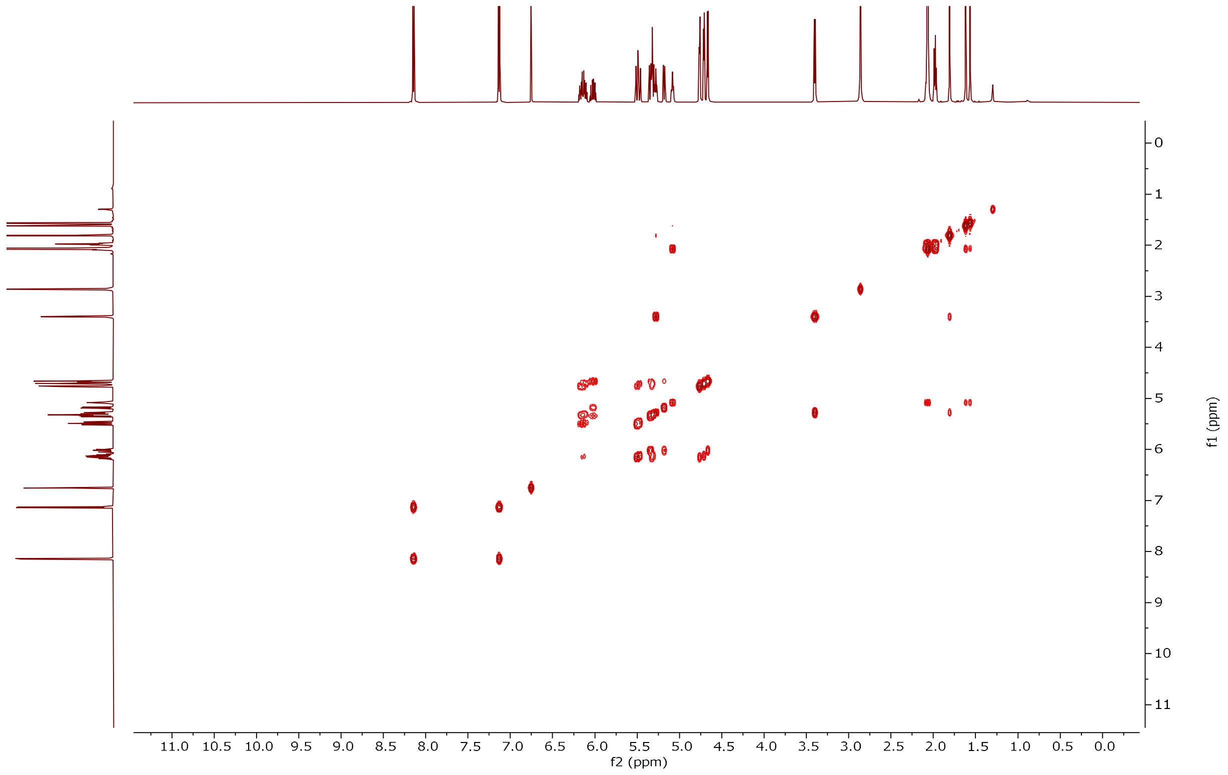

Figure S6: ^{1}H-^{1}H COSY spectrum of **6a**

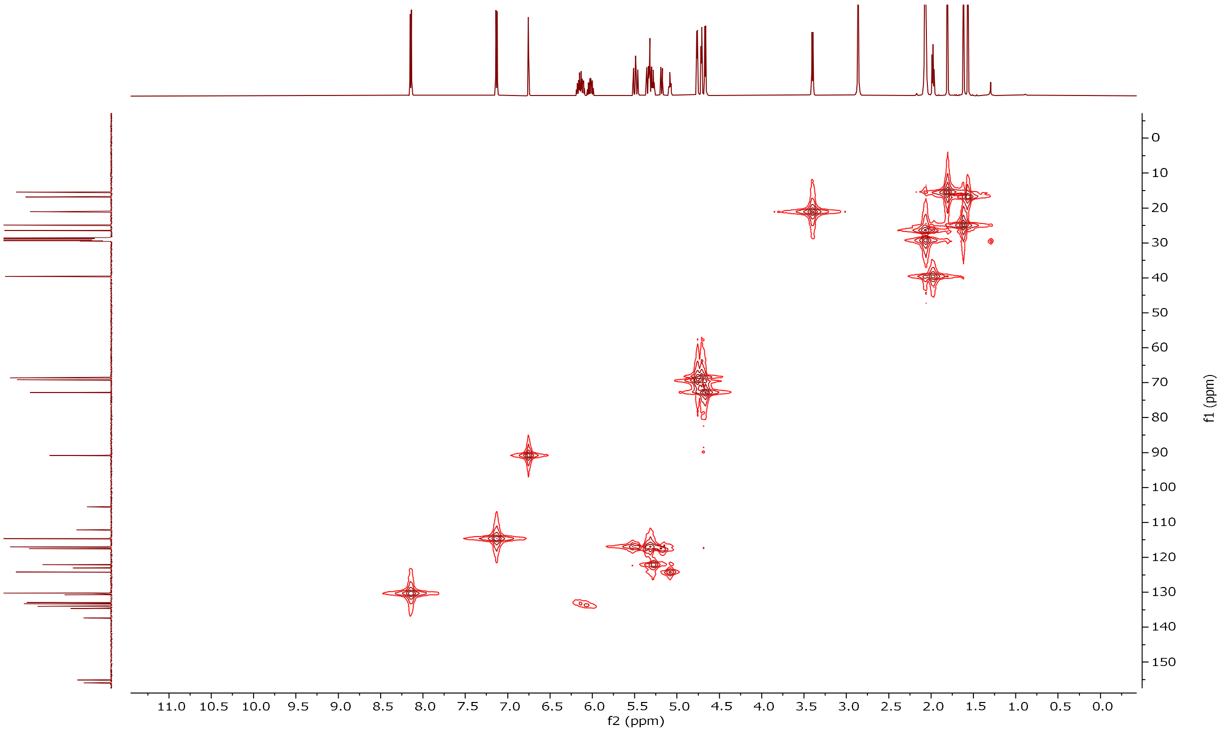

Figure S7: HSQC spectrum of **6a**

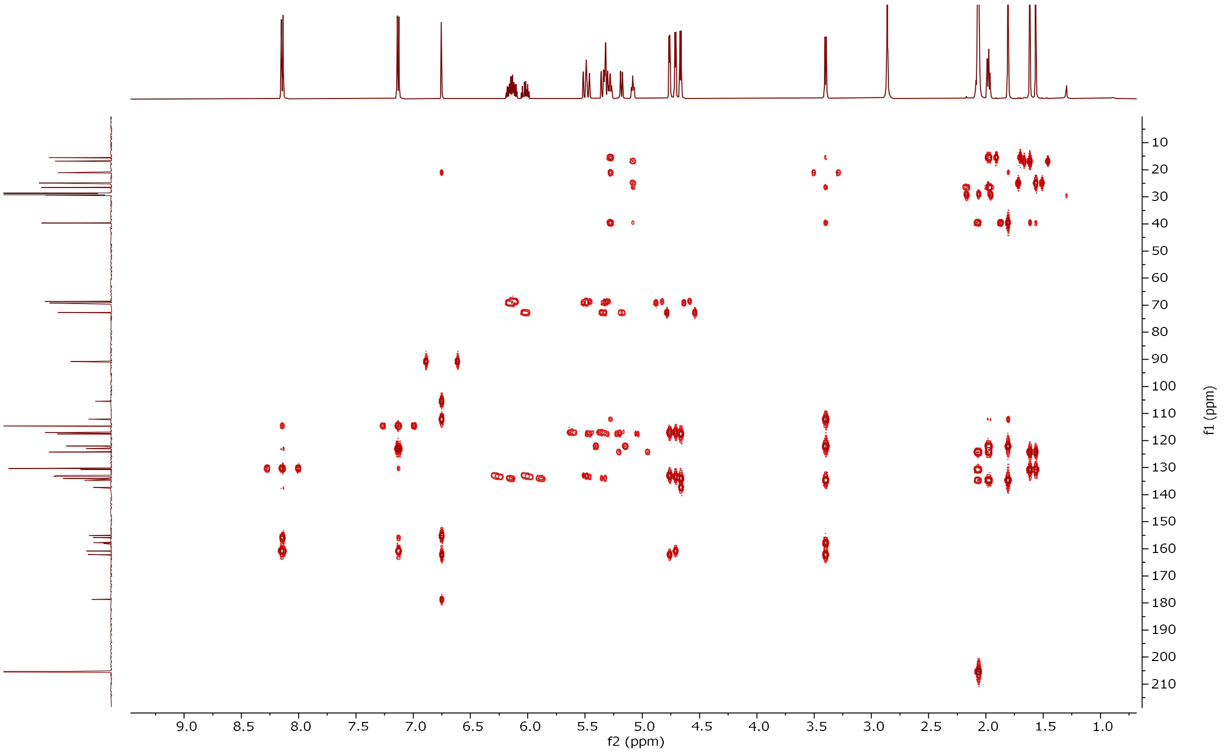

Figure S8: HMBC spectrum of **6a**

**Measured Ion Mass(es) :**	583.3052	**Deviation [mmu] :**	0.22 [mmu]
**Calculated Ion Mass(es) :**	583.30542	**Deviation [ppm] :**	0.38 [ppm]
**Potential Molecular Formula :**	C37H42O6H+		

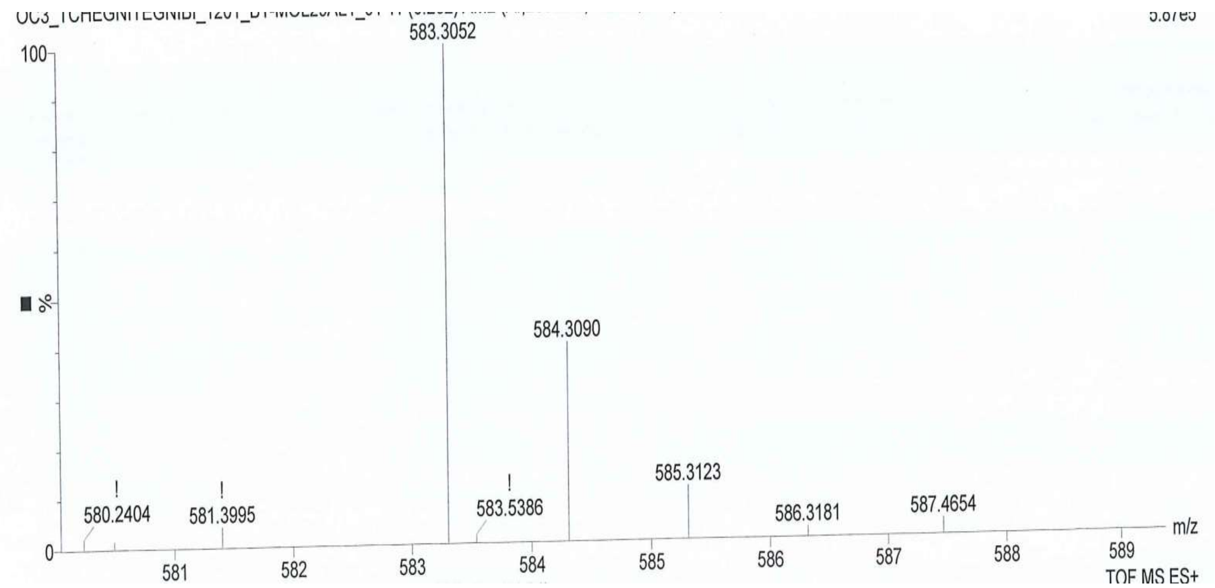

Figure S9: ESI-HR Mass spectrum of **6b**

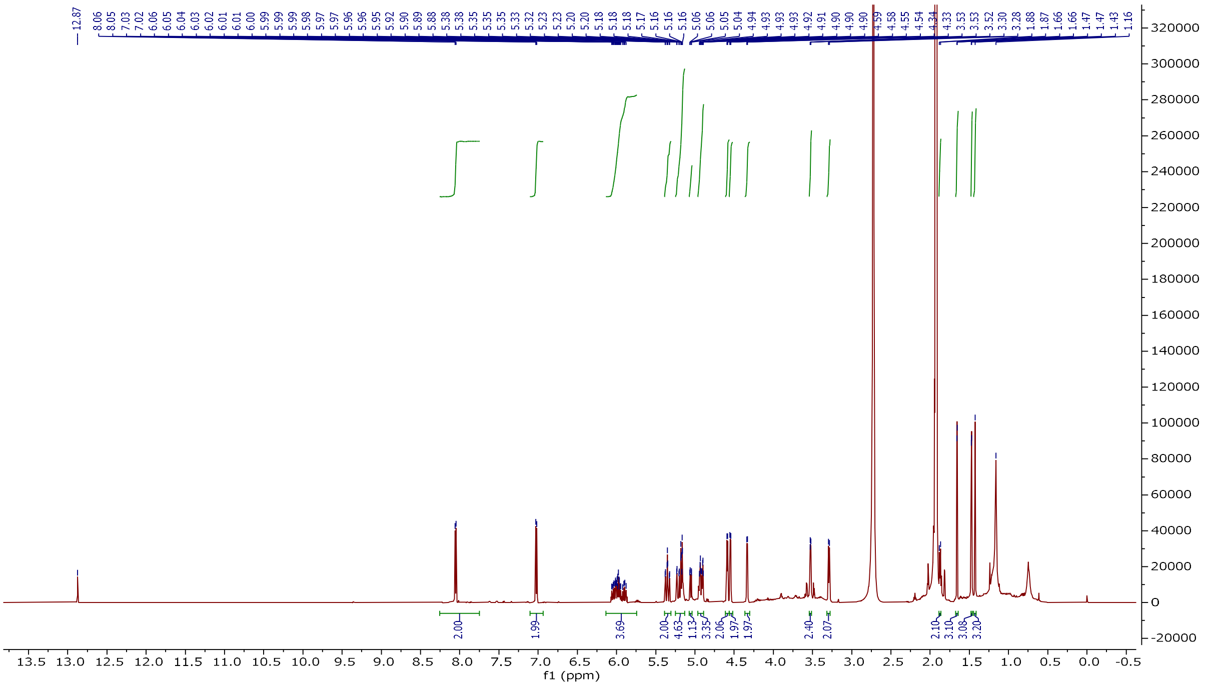

Figure S10: ^{1}H-NMR (500 MHz, acetone –d_6) spectrum of **6b**

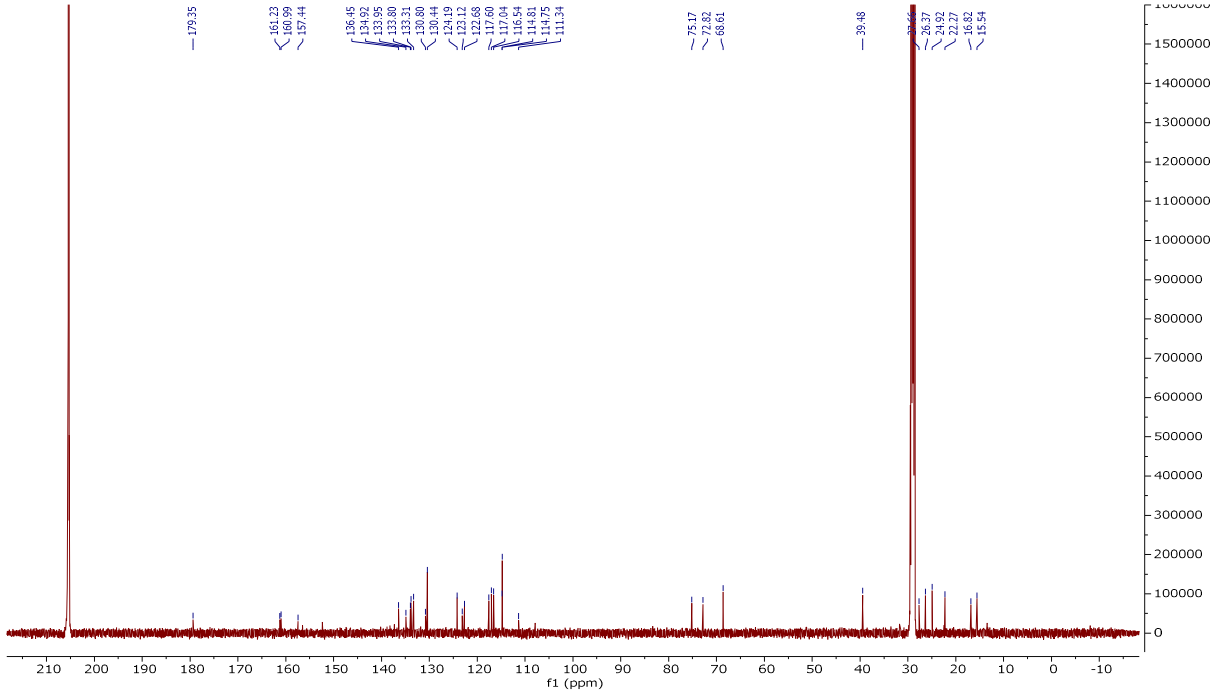

Figure S11: ^{13}C-NMR (125 MHz, acetone –d_6) spectrum of **6b**

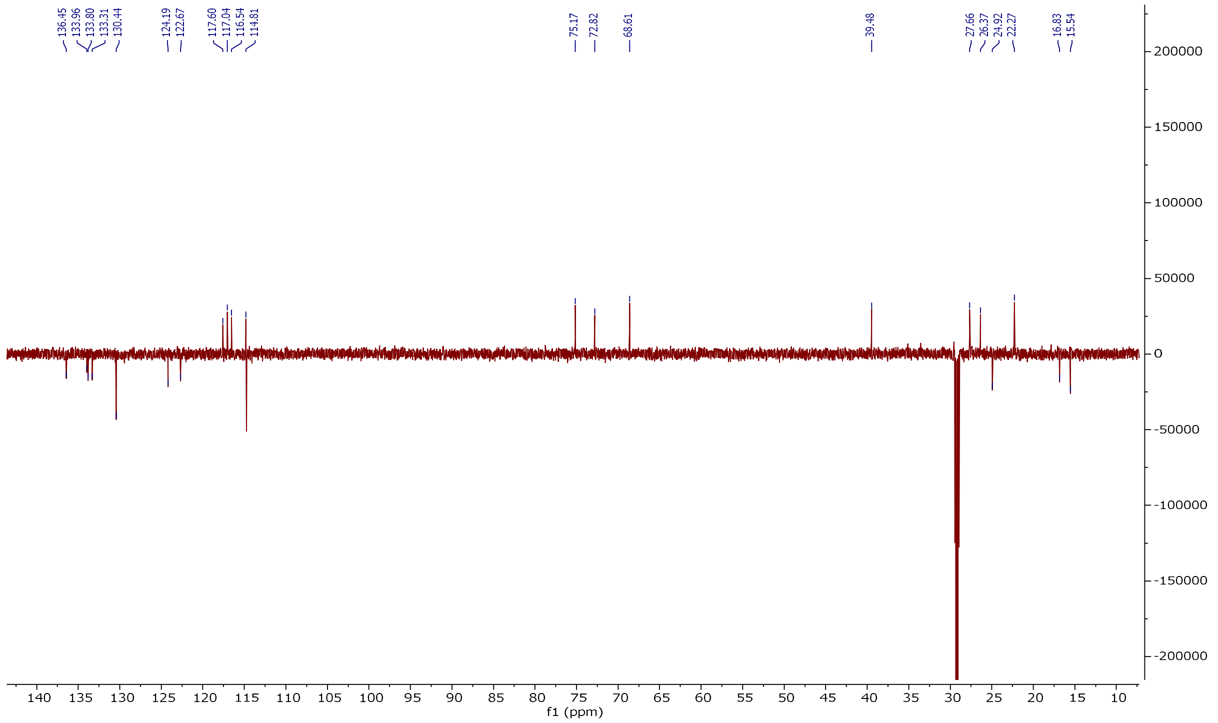

Figure S12: DEPT 135 spectrum of **6b**

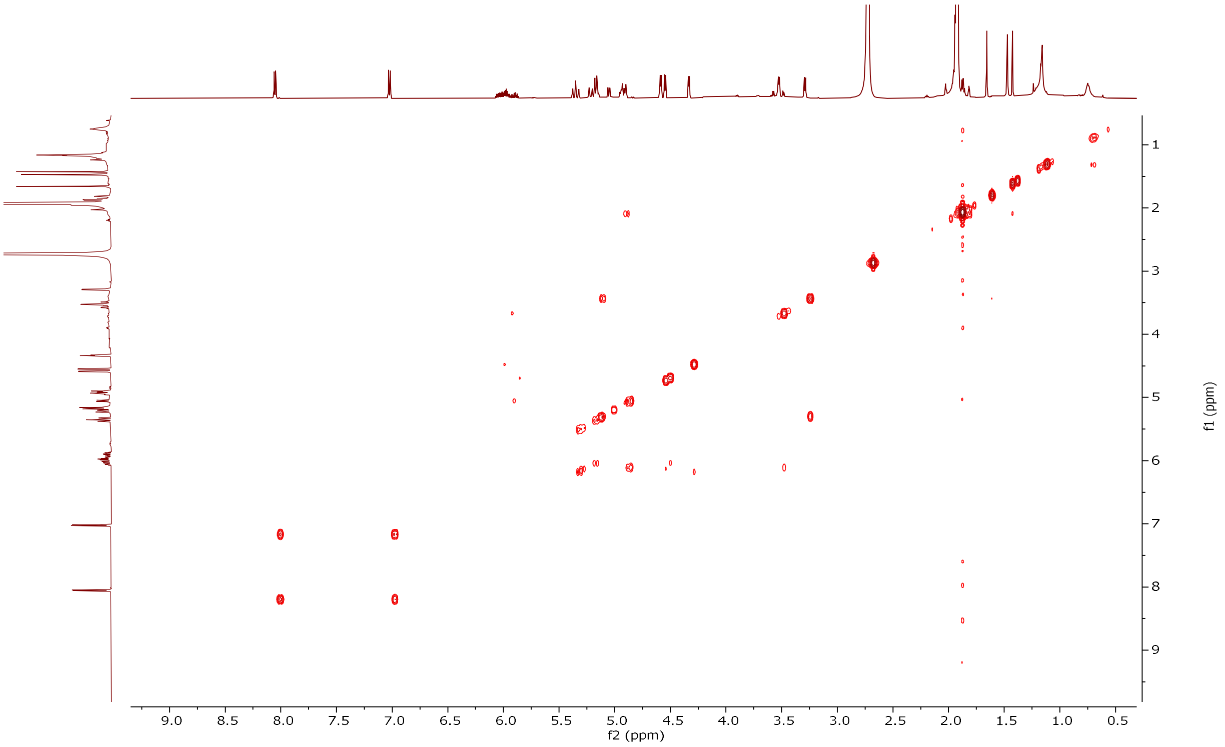

Figure S13: ¹H-¹H COSY spectrum of **6b**

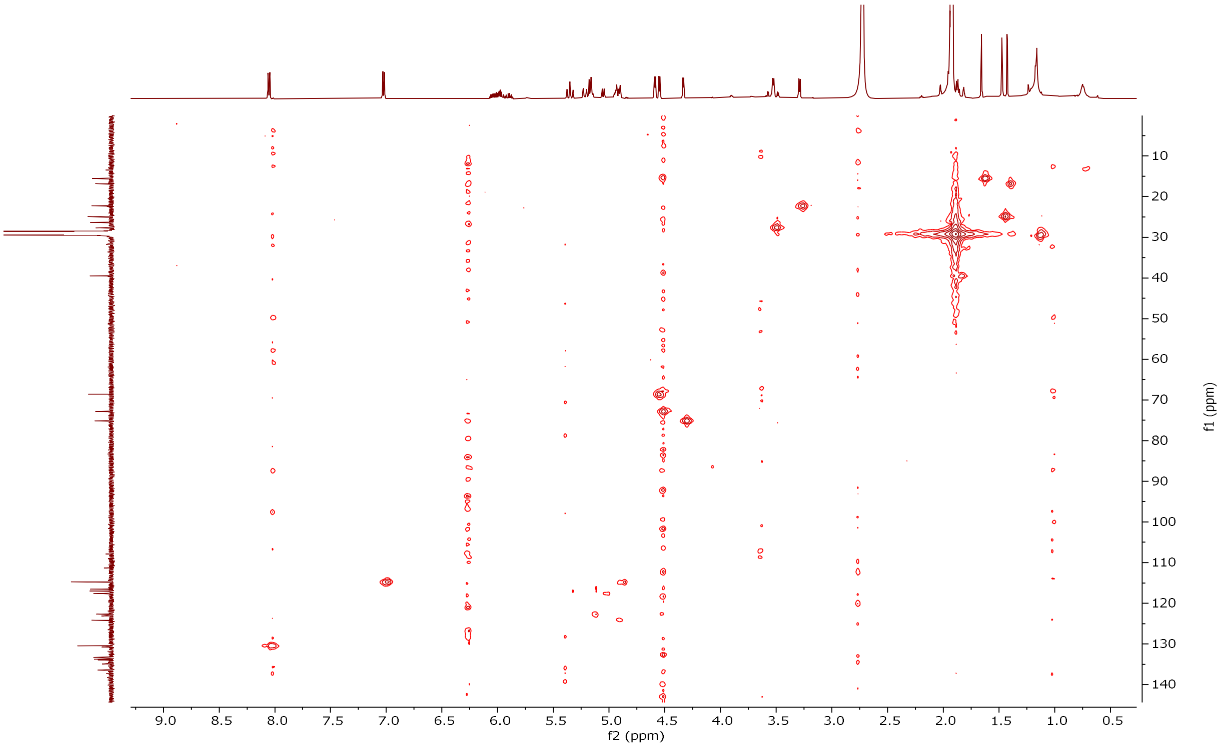

Figure S14: HSQC spectrum of **6b**

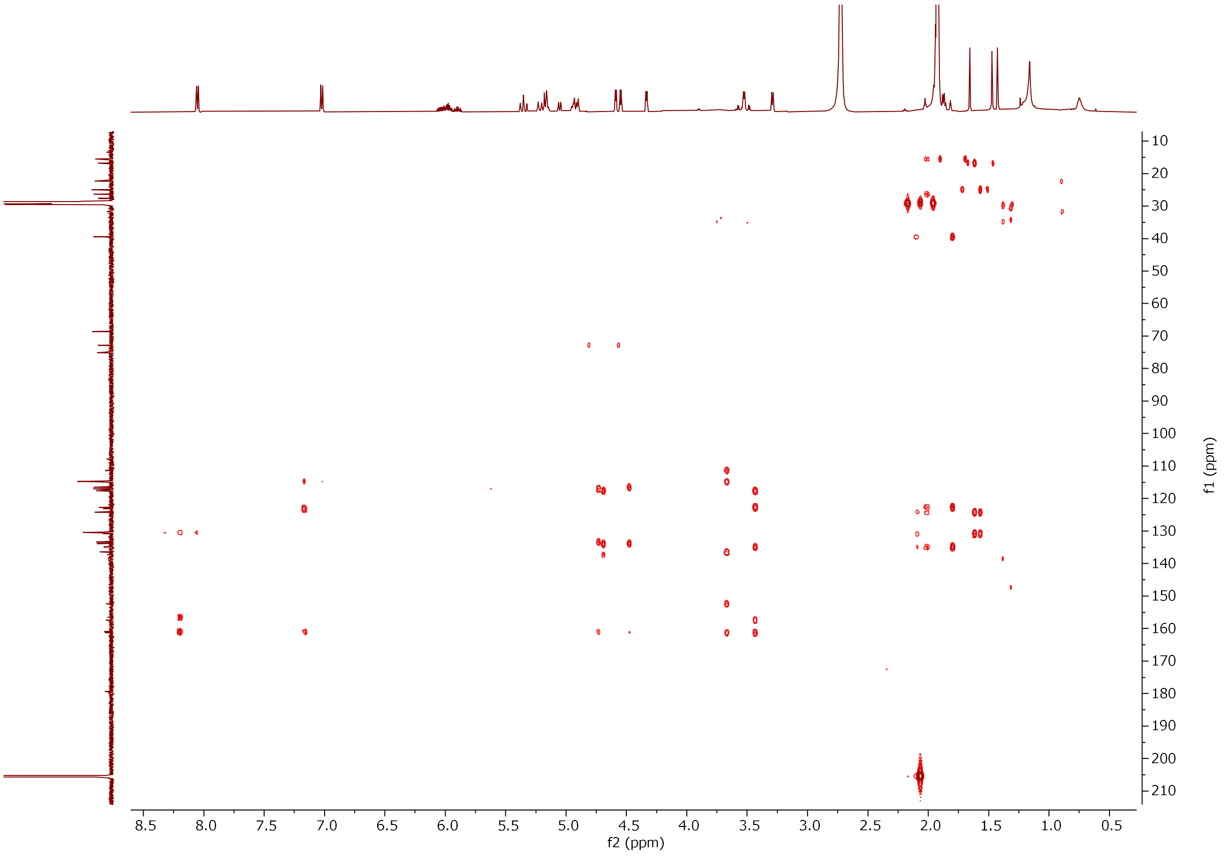

Figure S15: HMBC spectrum of **6b**

**Measured Ion Mass(es) :**	613.2043	**Deviation [mmu] :**	0.12 [mmu]
**Calculated Ion Mass(es) :**	613.20442	**Deviation [ppm] :**	0.20 [ppm]
**Potential Molecular Formula :**	C33H34O10Na+		

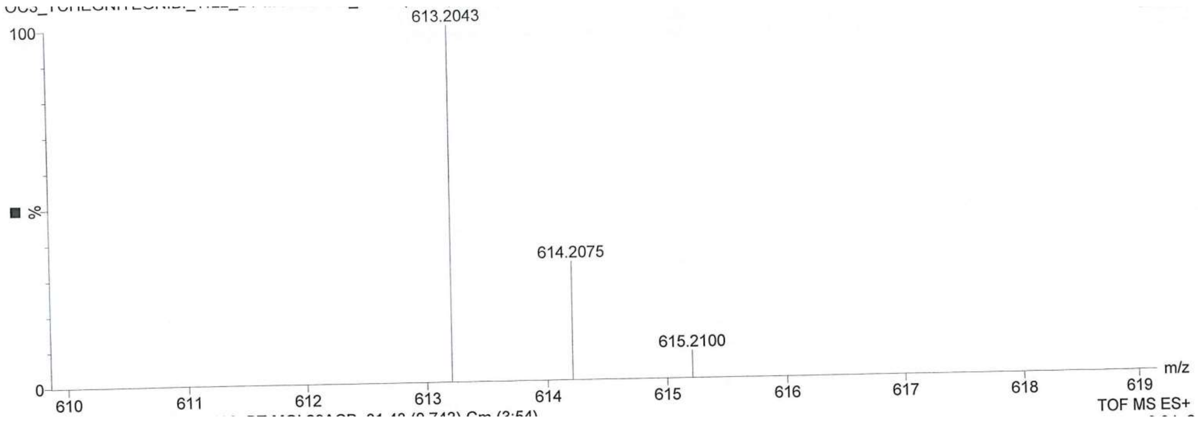

Figure S16: ESI-HR Mass spectrum of **6c**

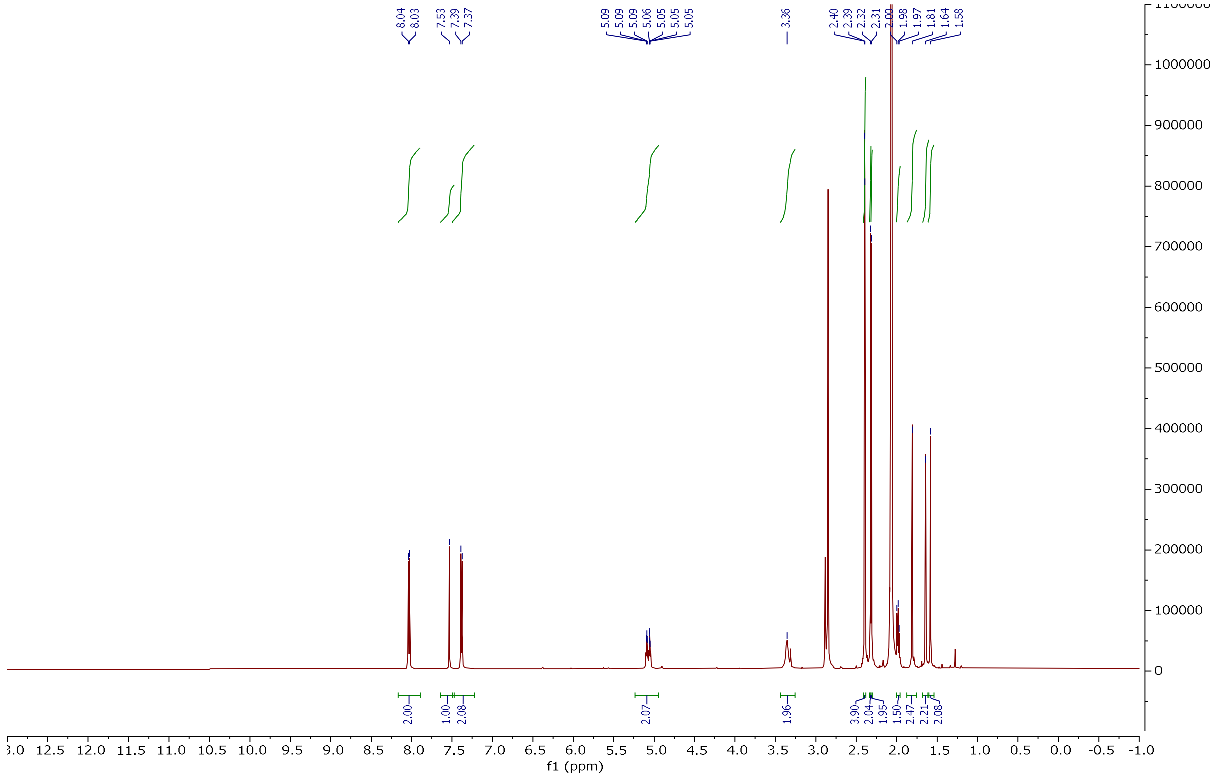

Figure S17: ^{1}H-NMR (500 MHz, acetone –d_6) spectrum of **6c**

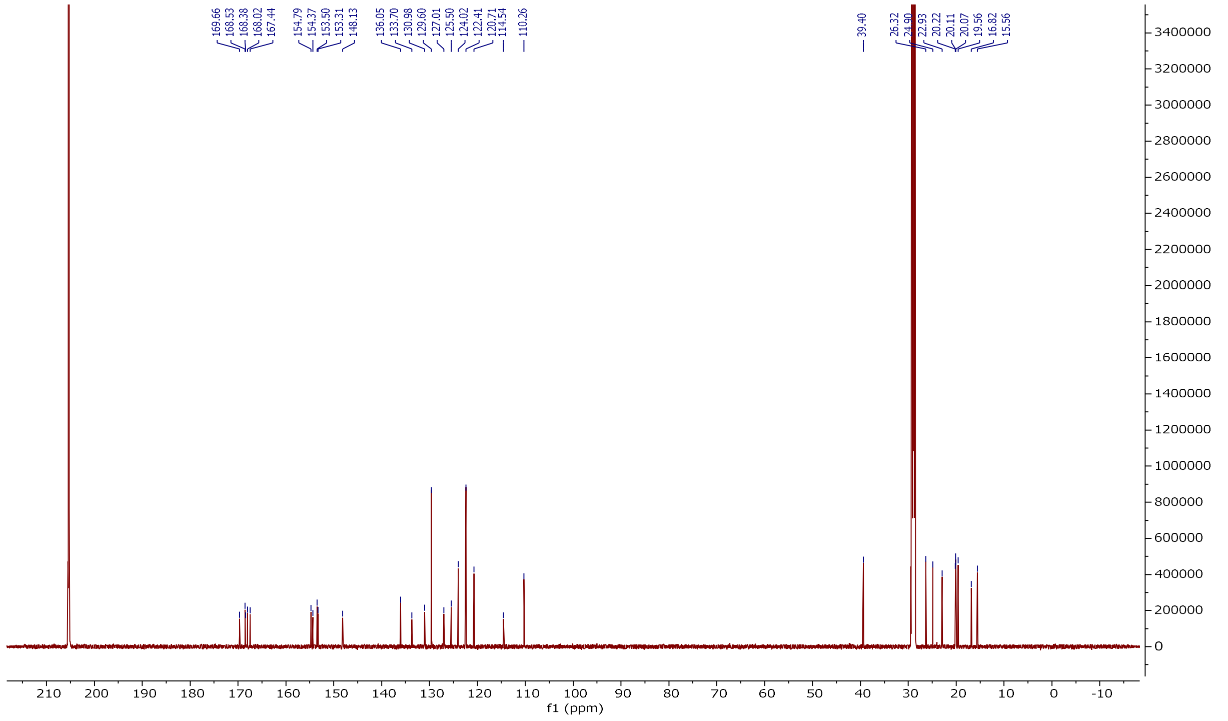

Figure S18: ^{13}C-NMR (125 MHz, acetone –d_6) spectrum of **6c**

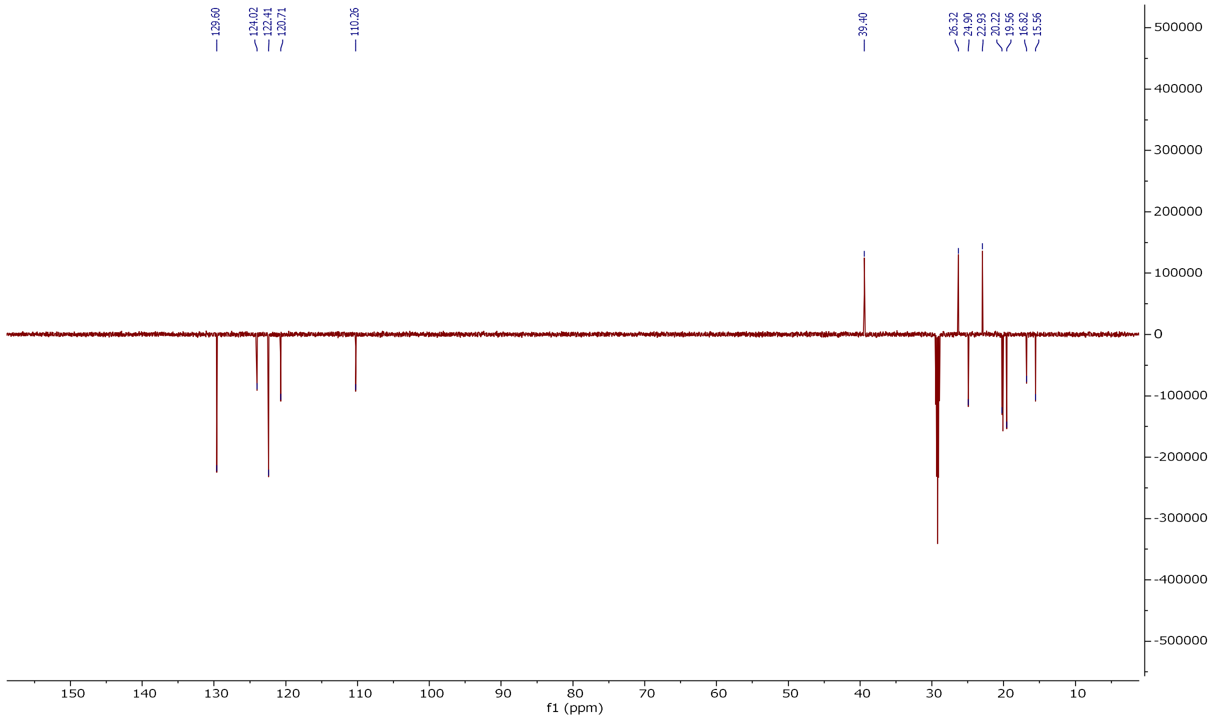

Figure S19: DEPT 135 spectrum of **6c**

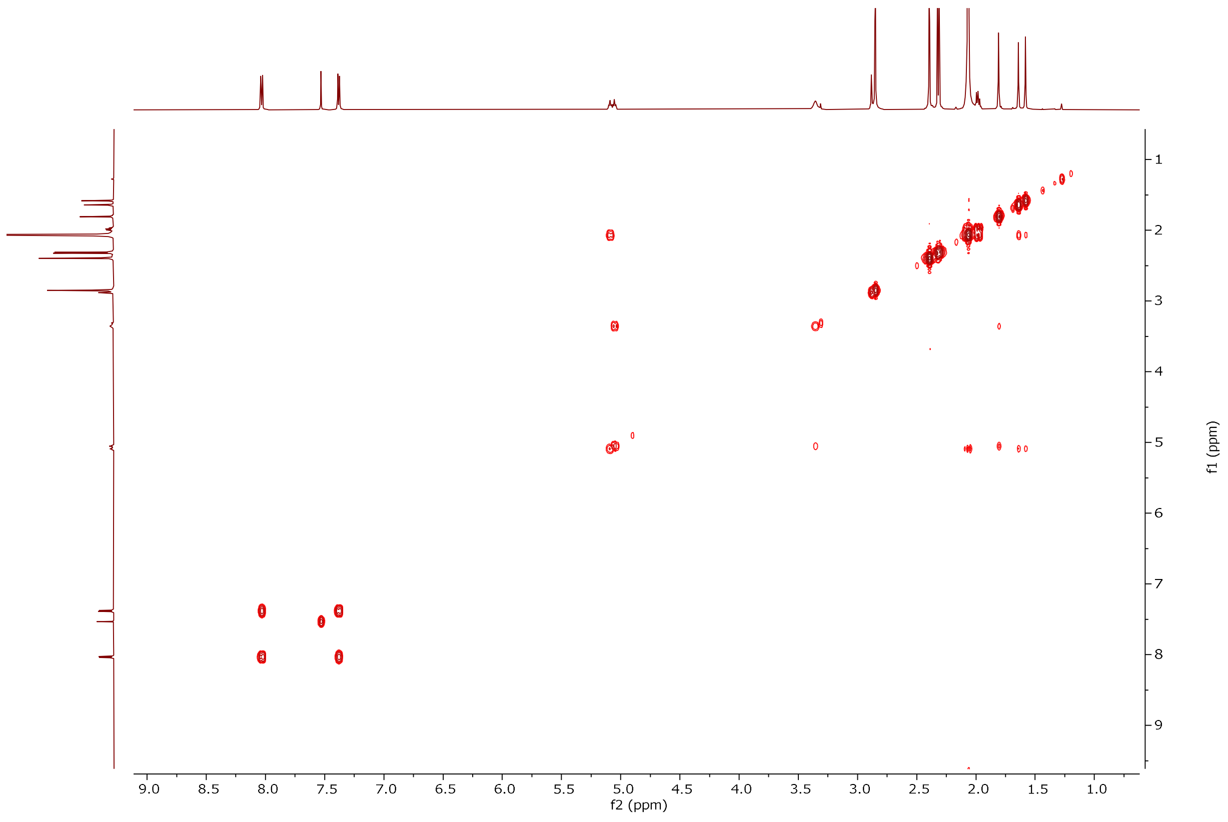

Figure S20: ^{1}H-^{1}H COSY spectrum of **6c**

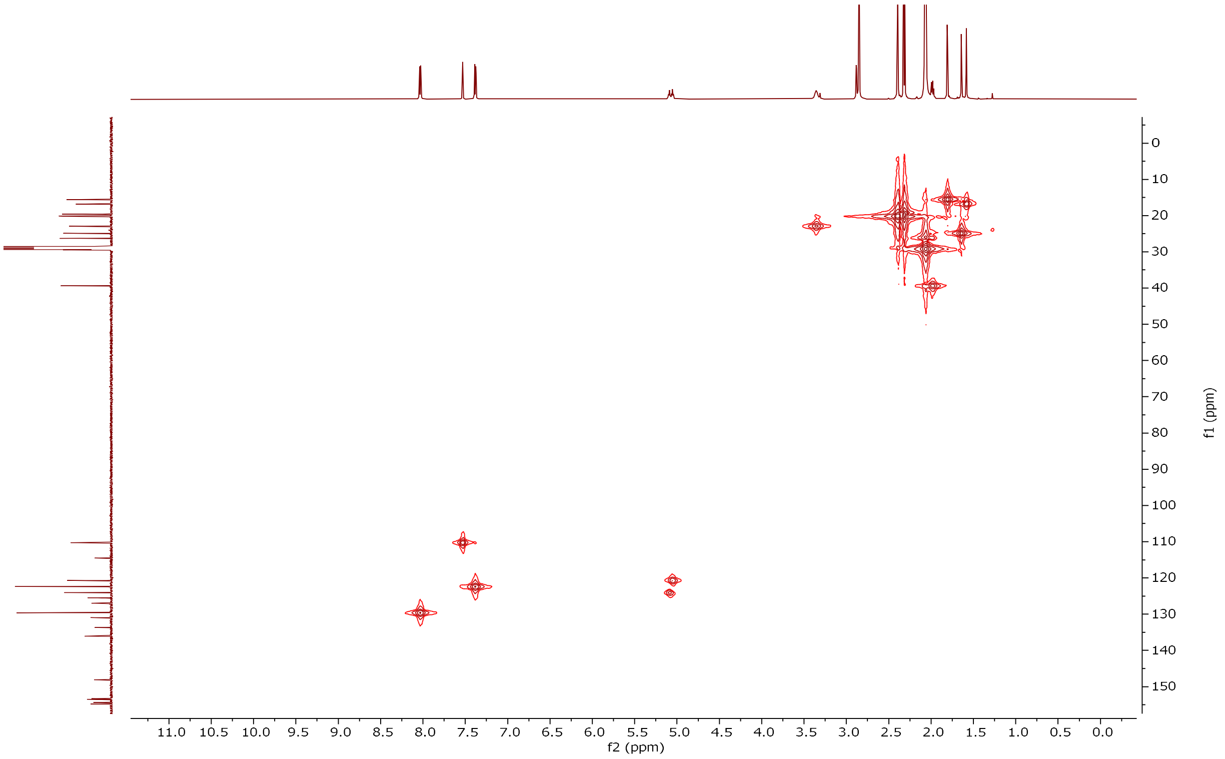

Figure S21: HSQC spectrum of **6c**

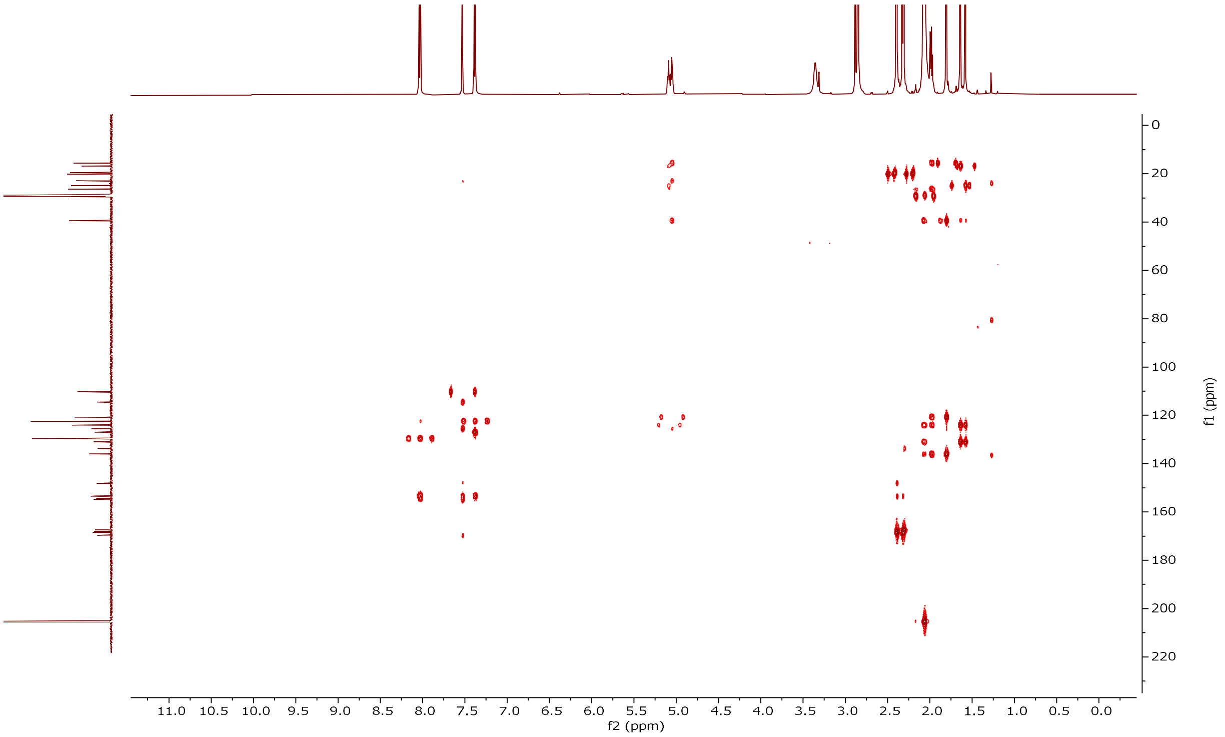

Figure S22: HMBC spectrum of **6c**

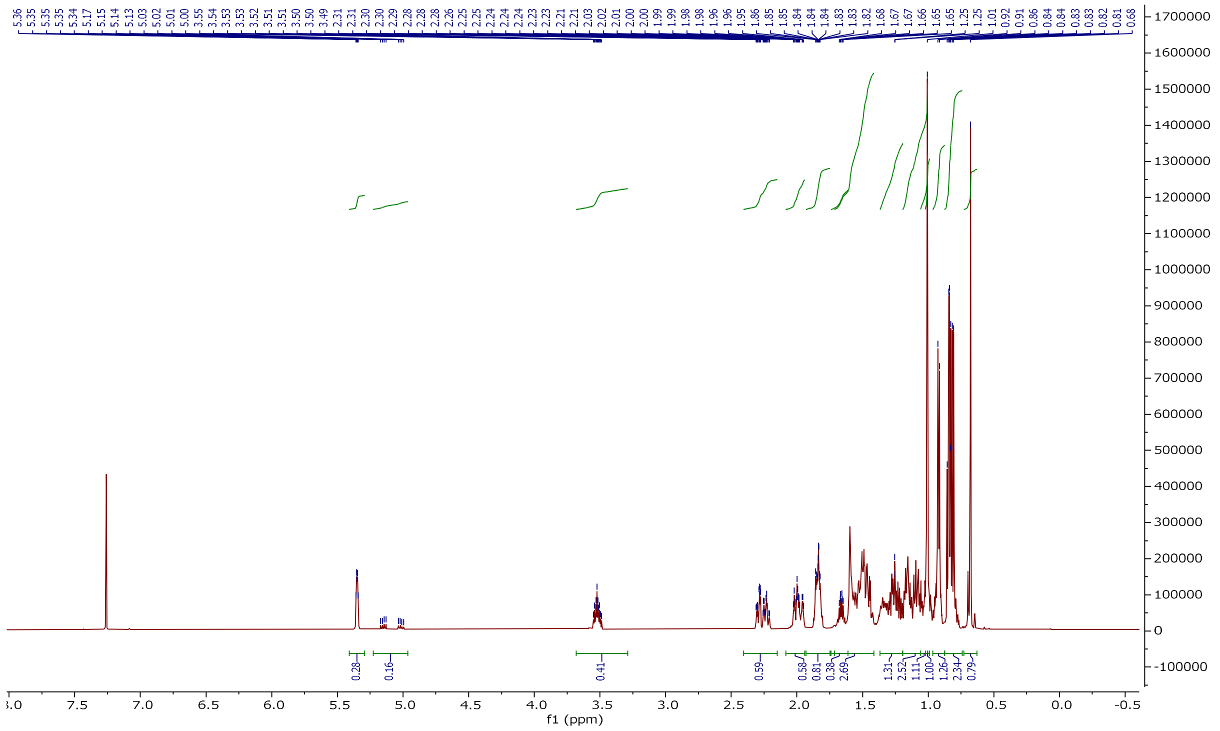

Figure S23: ^{1}H-NMR (500 MHz, $CDCl_3$) spectrum of **1+2**

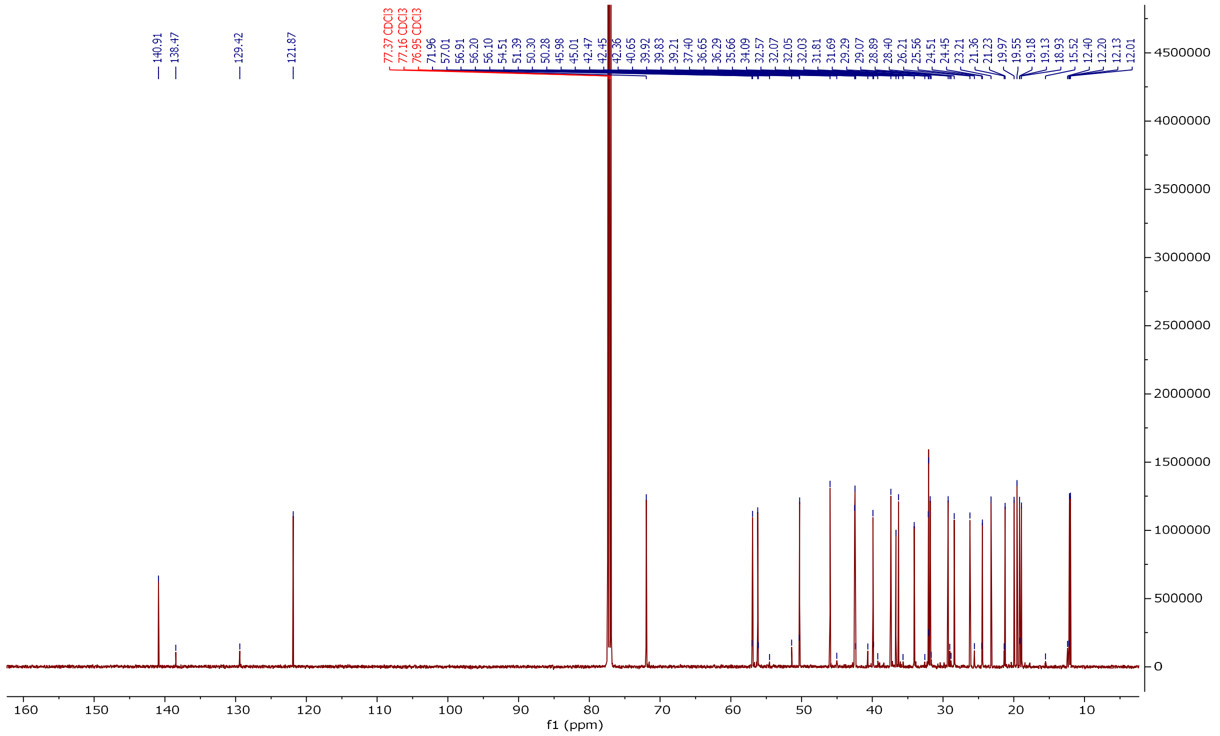

Figure S24: ^{13}C-NMR (125 MHz, $CDCl_3$) spectrum of **1+2**

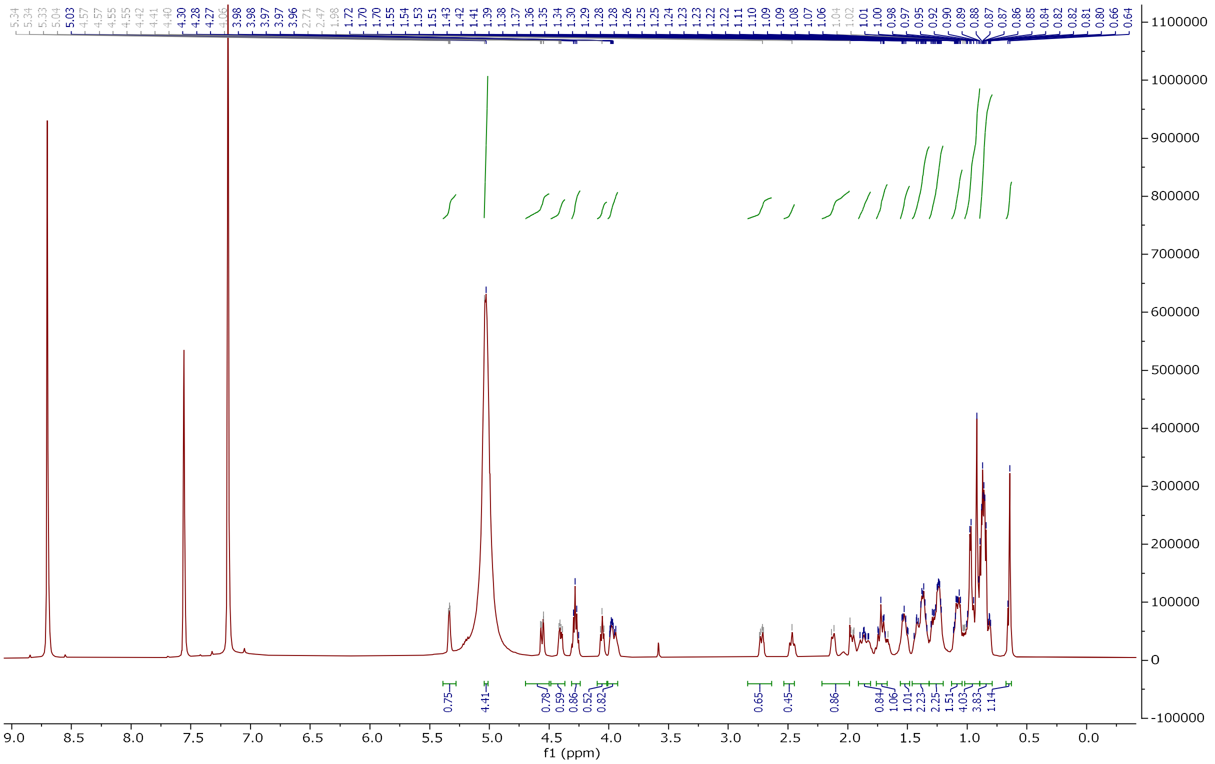

Figure S25: ^{1}H-NMR (500 MHz, Pyridine–d_5) spectrum of **3**

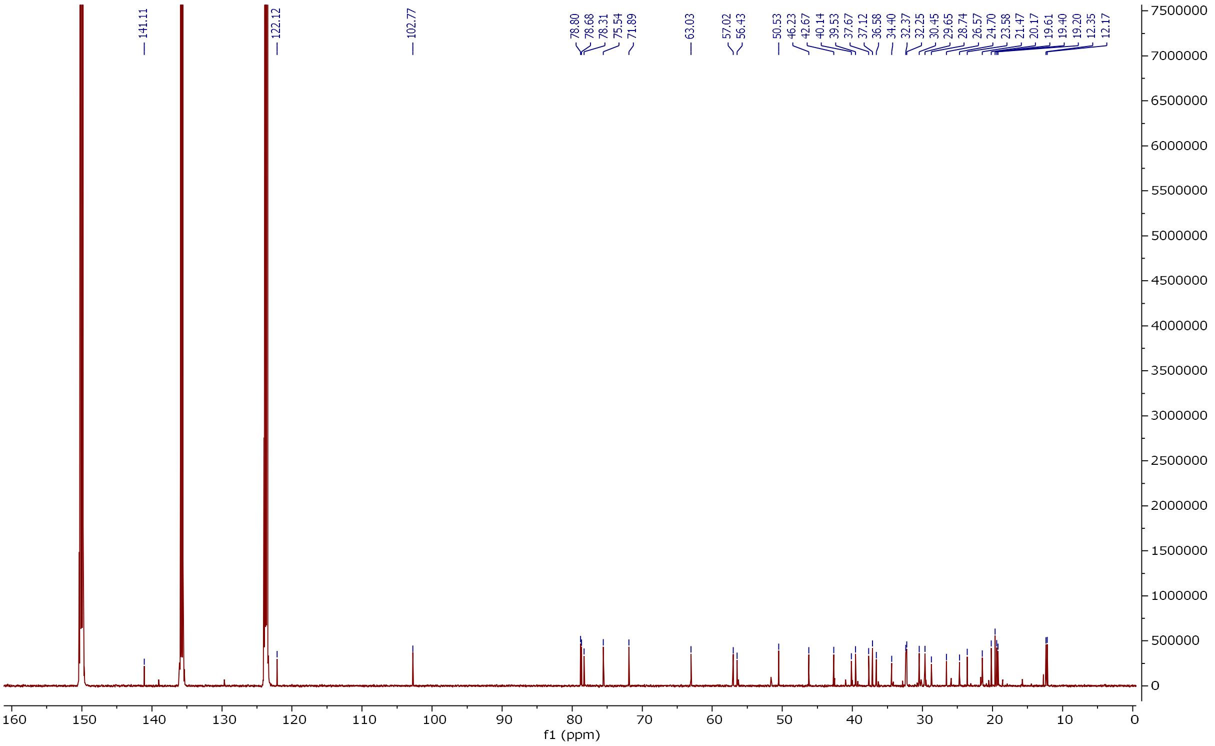

Figure S26: ^{13}C-NMR (125 MHz, Pyridine–d_5) spectrum of **3**

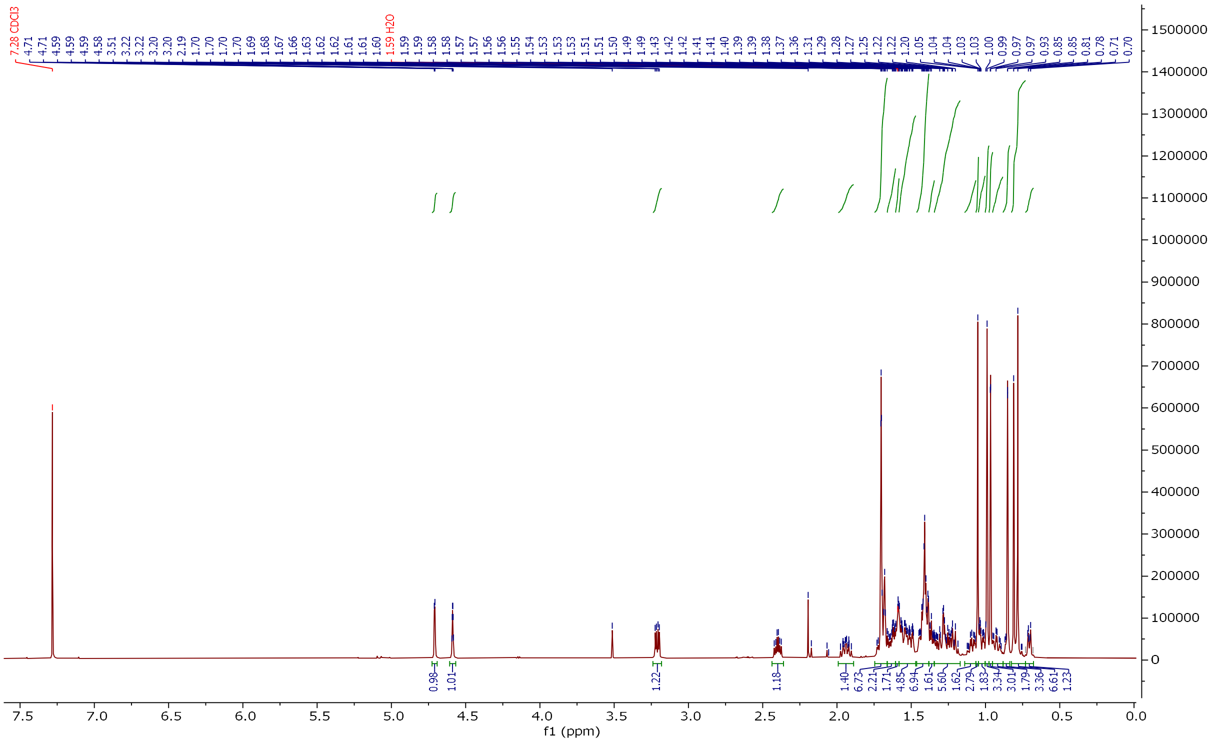

Figure S27: ^{1}H-NMR (500 MHz, $CDCl_3$) spectrum of **4**

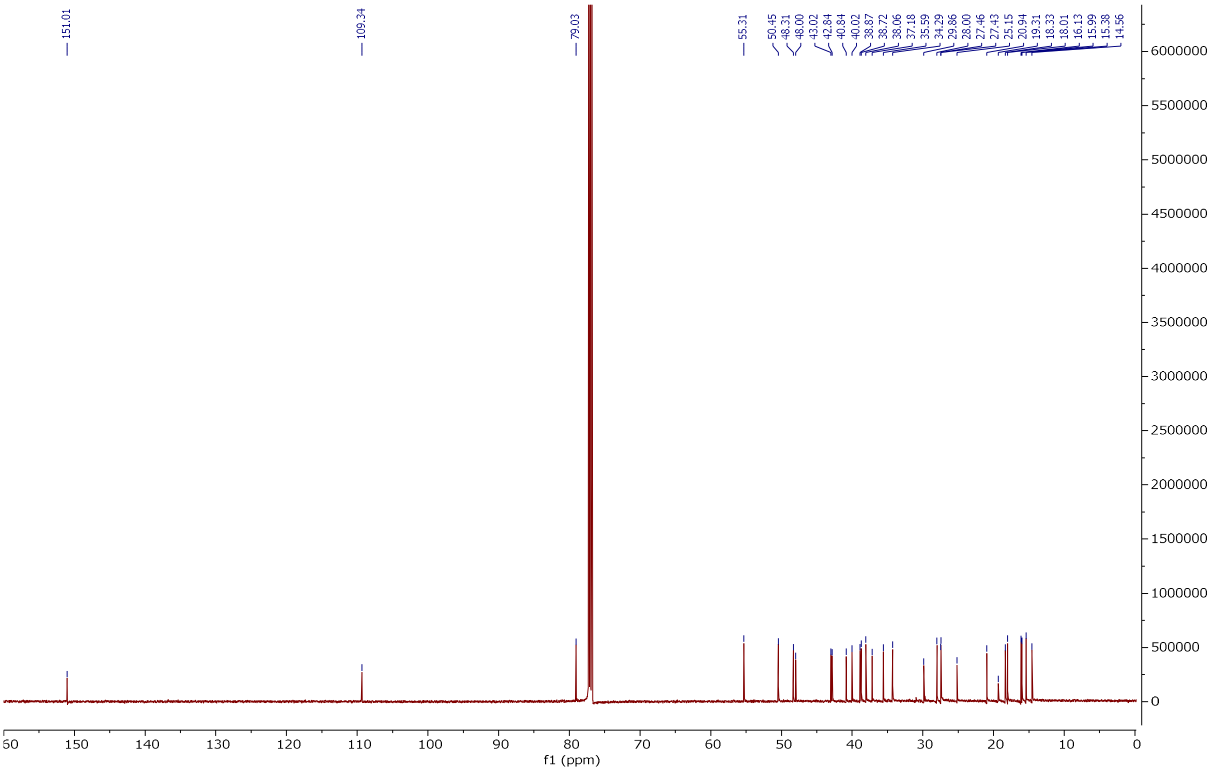

Figure S28: ^{13}C-NMR (125 MHz, $CDCl_3$) spectrum of **4**

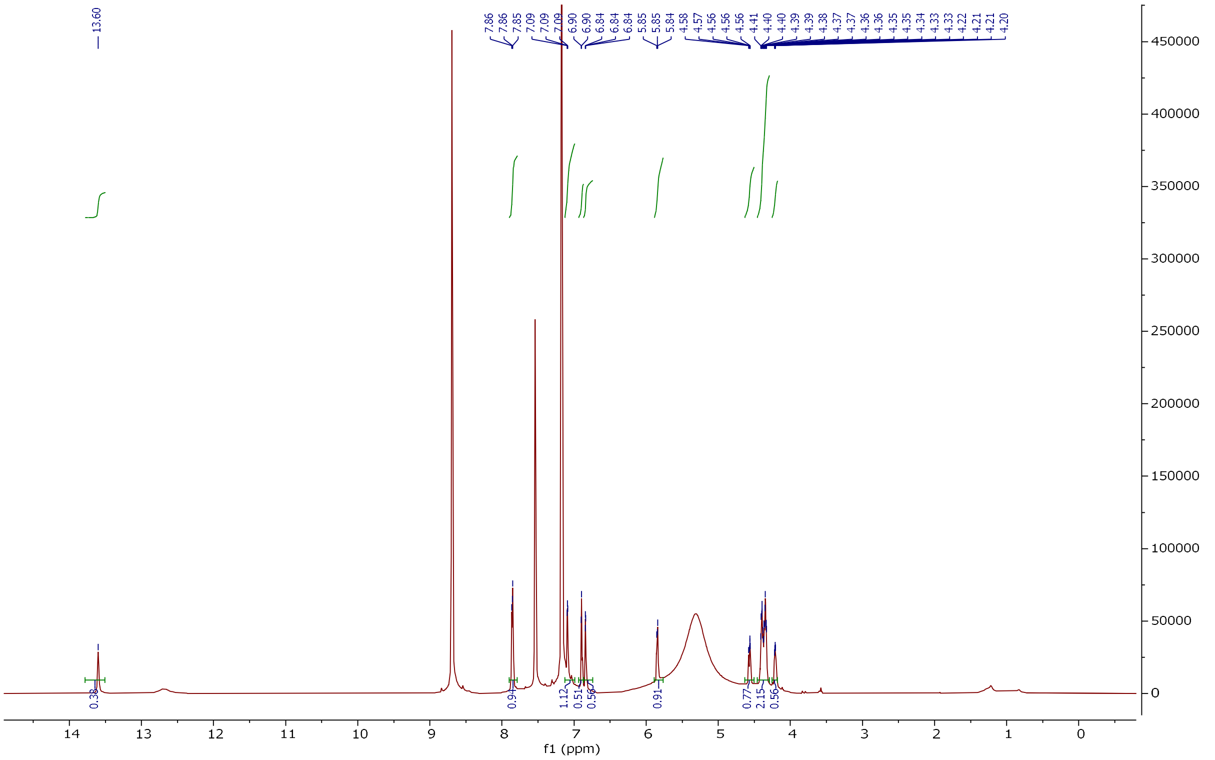

Figure S29: ^{1}H-NMR (500 MHz, acetone $-d_6$) spectrum of **5**

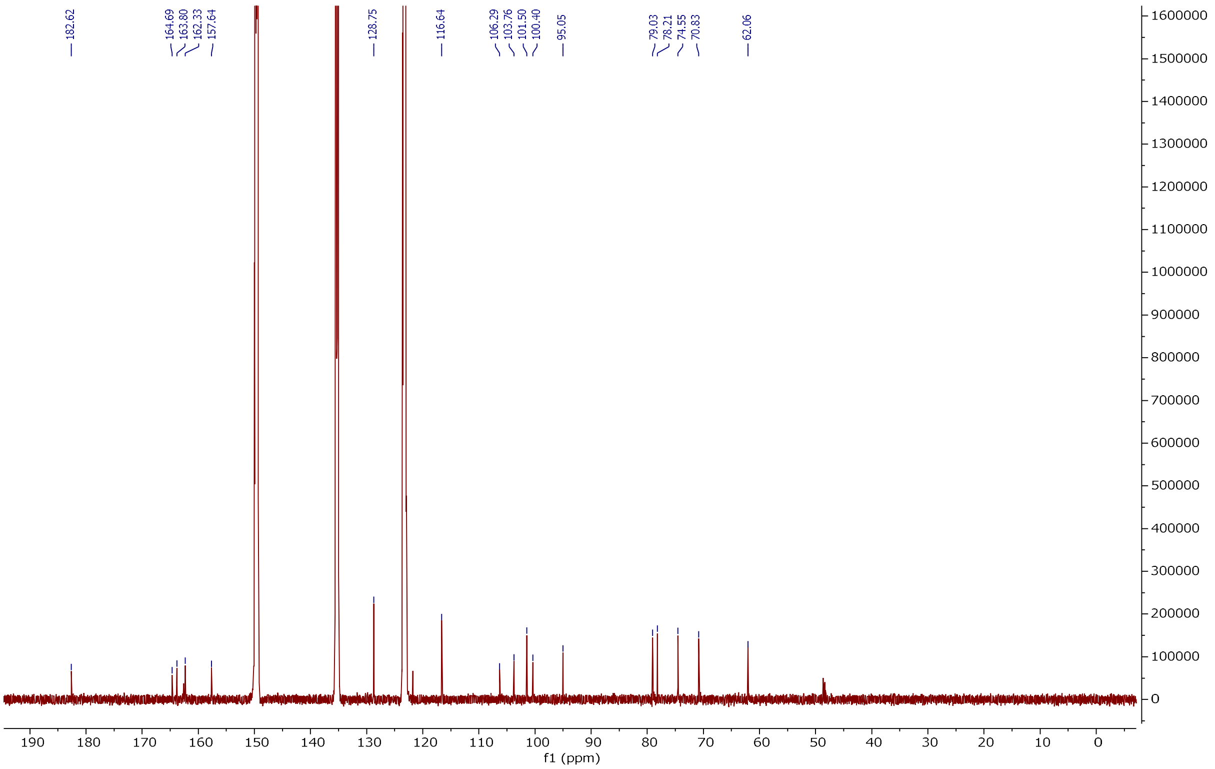

Figure S30: ^{13}C-NMR (125 MHz, acetone $-d_6$) spectrum of **5**

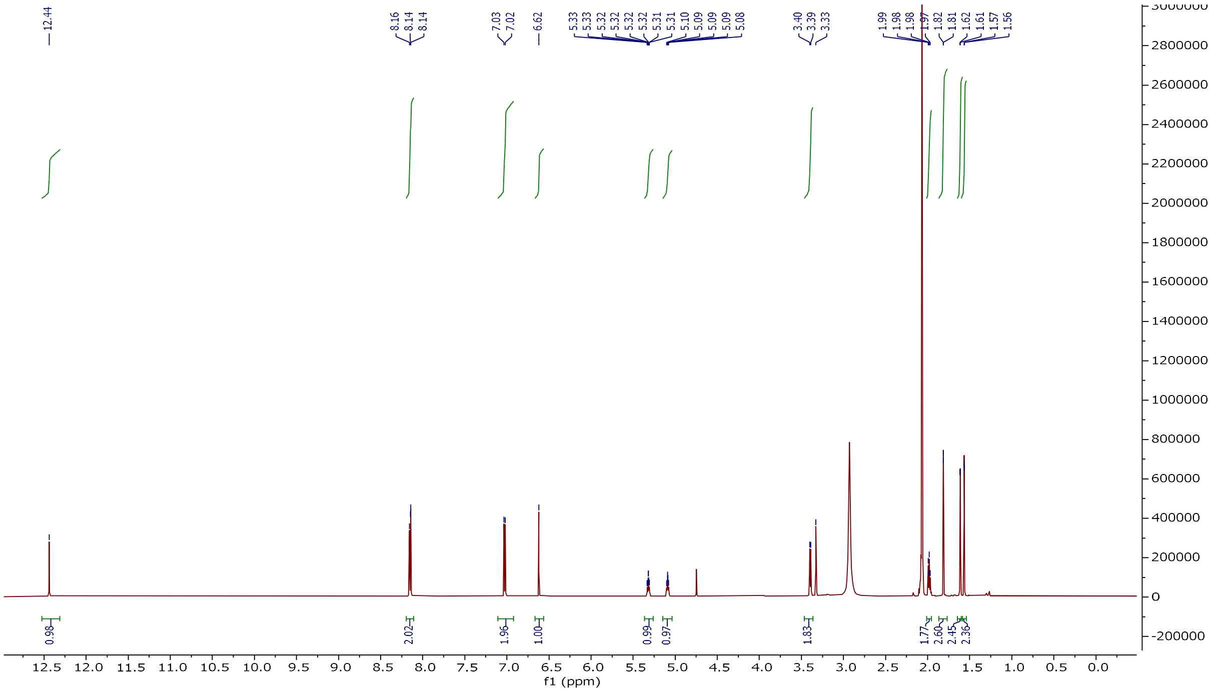

Figure S31: ^{1}H-NMR (500 MHz, acetone –d_6) spectrum of **6**

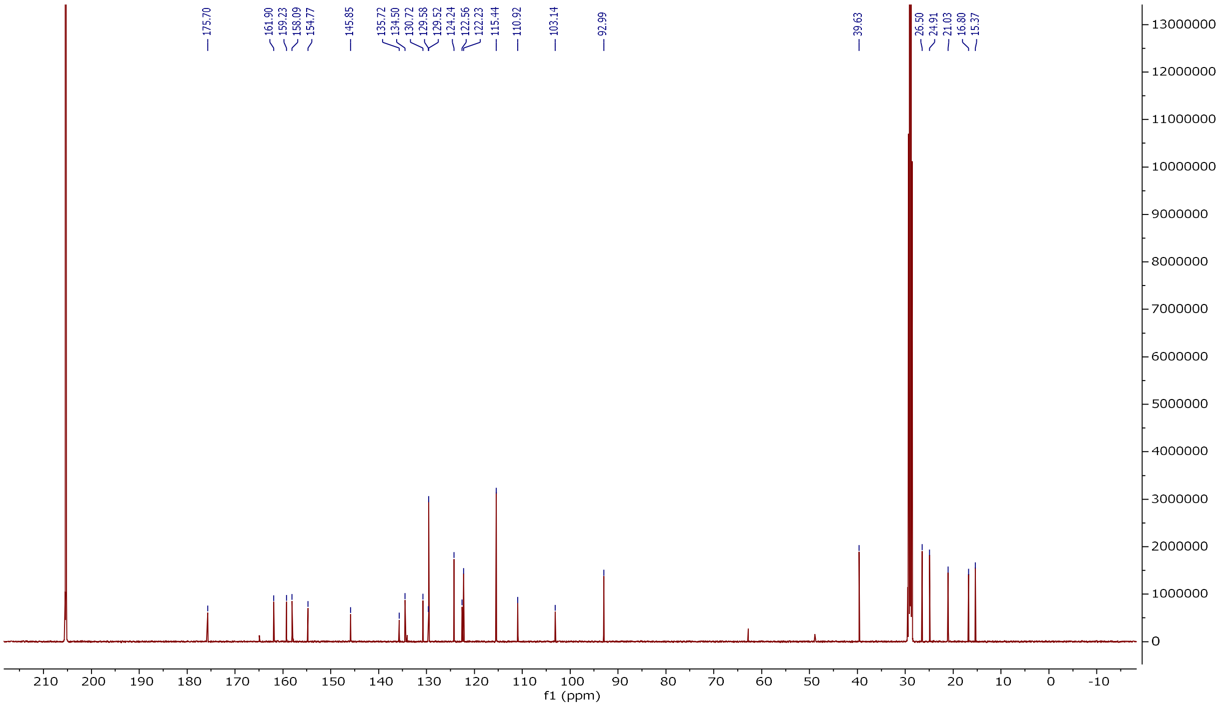

Figure S32: ^{13}C-NMR (1254 MHz, acetone –d_6) spectrum of **6**

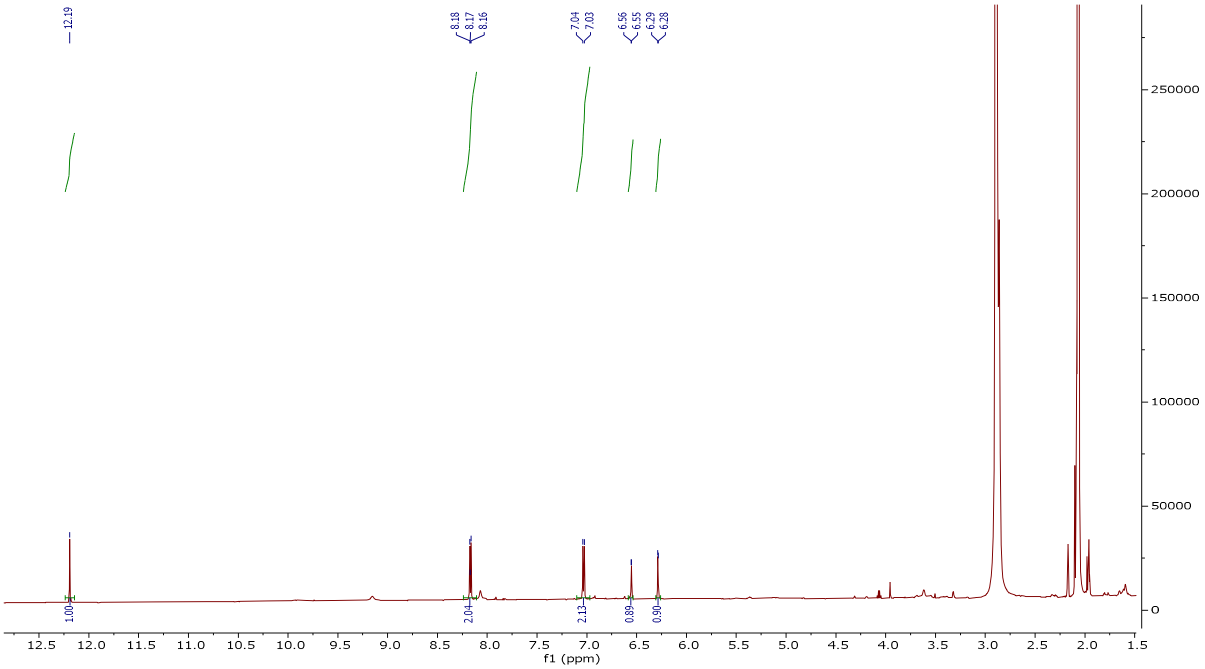

Figure S33: ^{1}H-NMR (500 MHz, acetone –d_6) spectrum of **7**

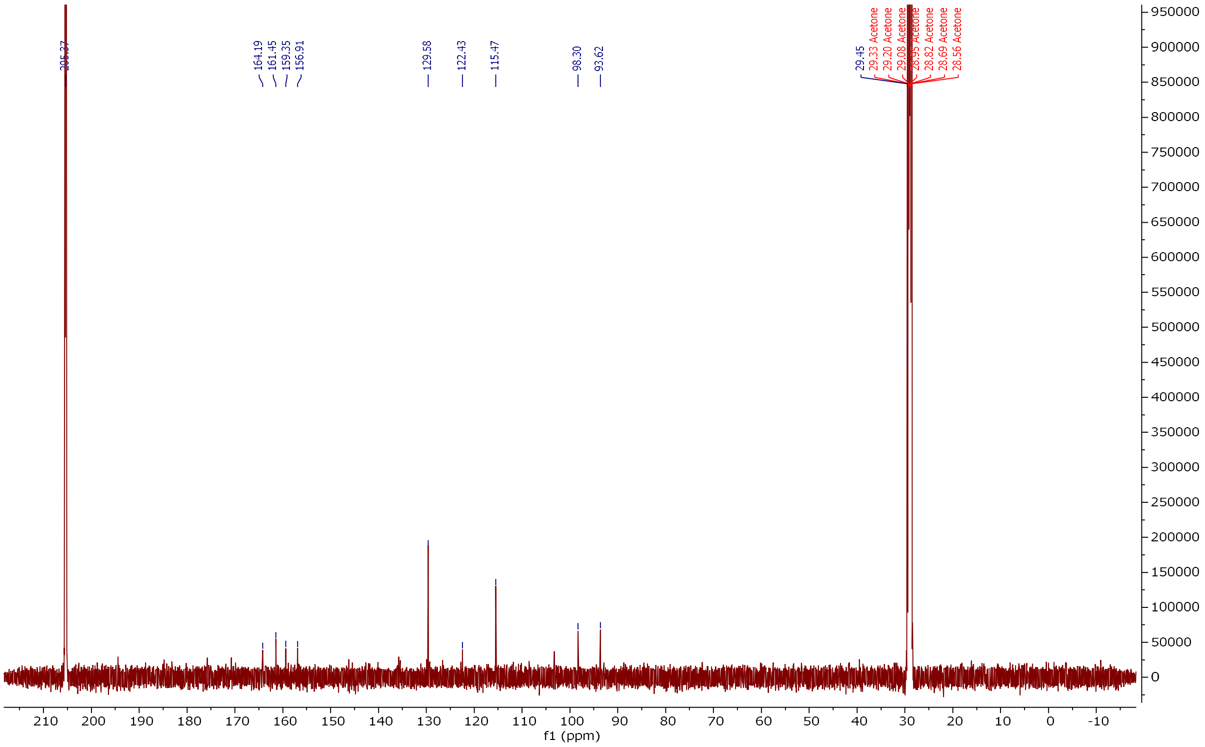

Figure S34: ^{13}C-NMR (1254 MHz, acetone –d_6) spectrum of **7**

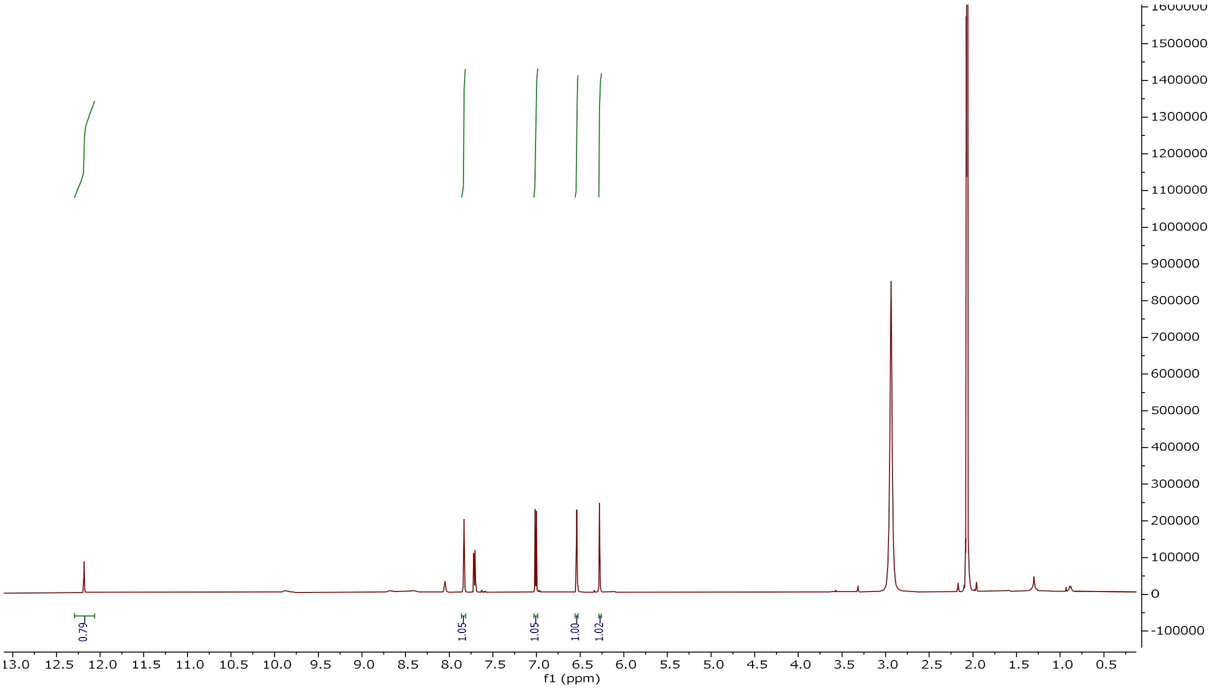

Figure S35: ^{1}H-NMR (500 MHz, acetone –*d6*) spectrum of **8**

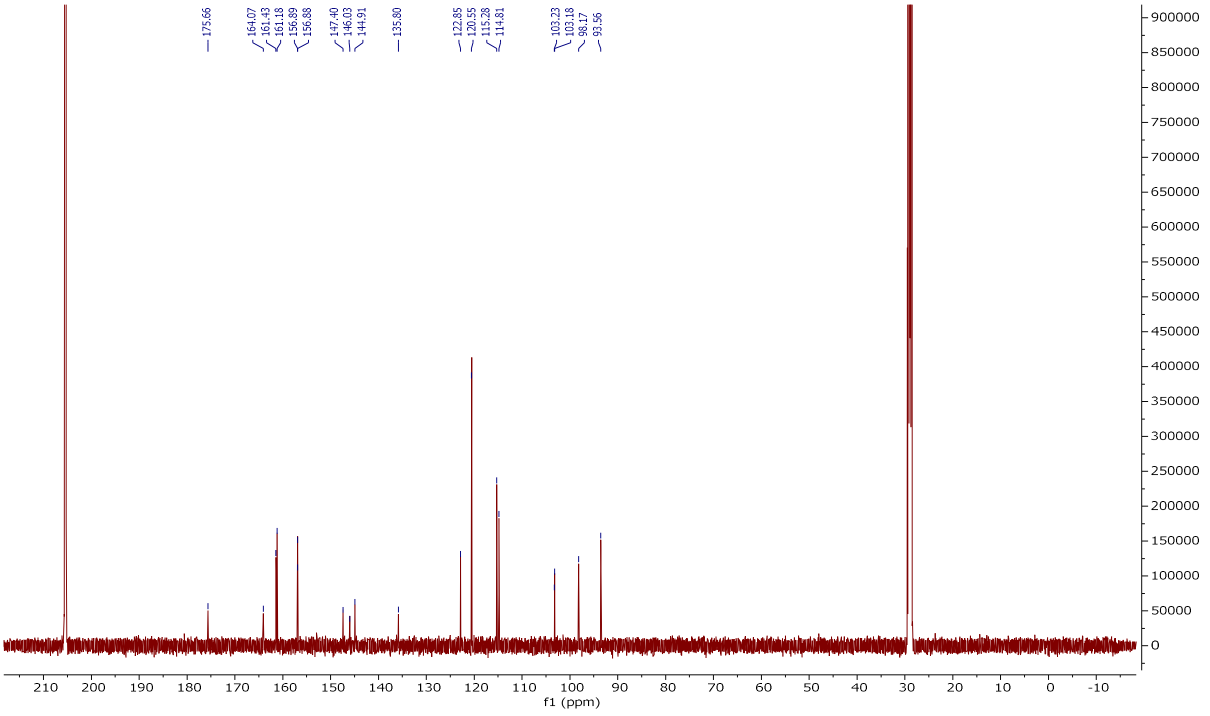

Figure S36: ^{13}C-NMR (1254 MHz, acetone –*d6*) spectrum of **8**

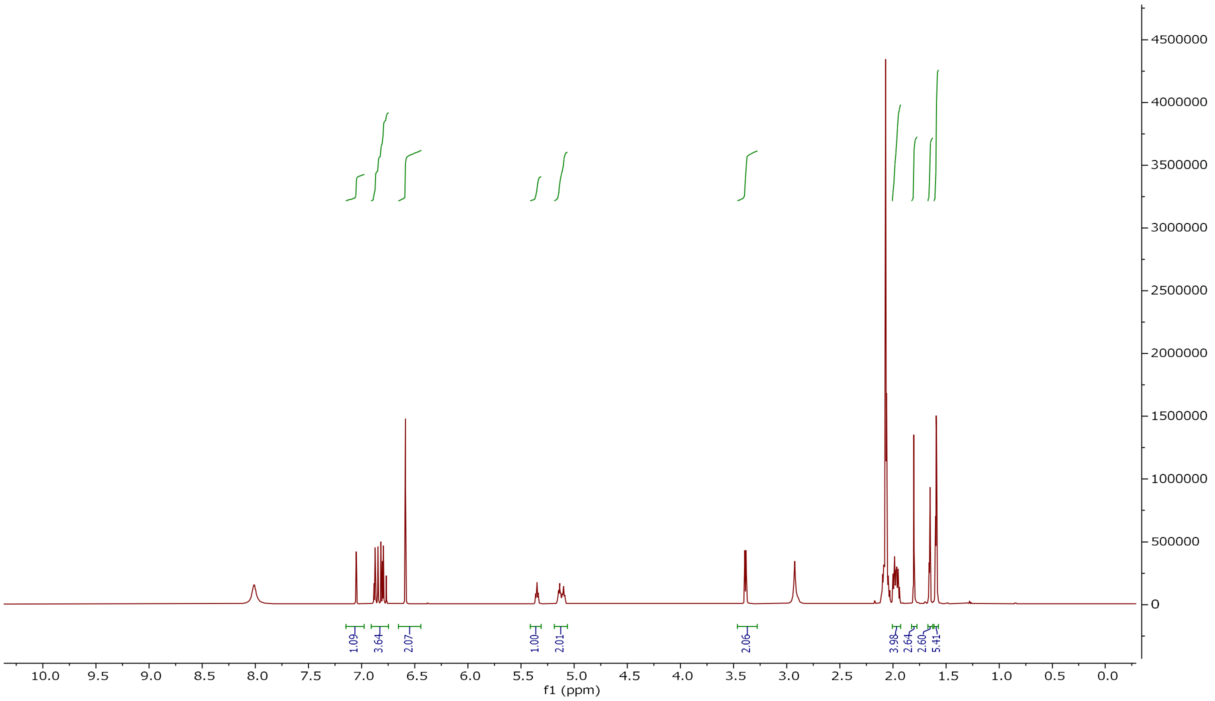

Figure S37: ^{1}H-NMR (500 MHz, acetone –*d*6) spectrum of **9**

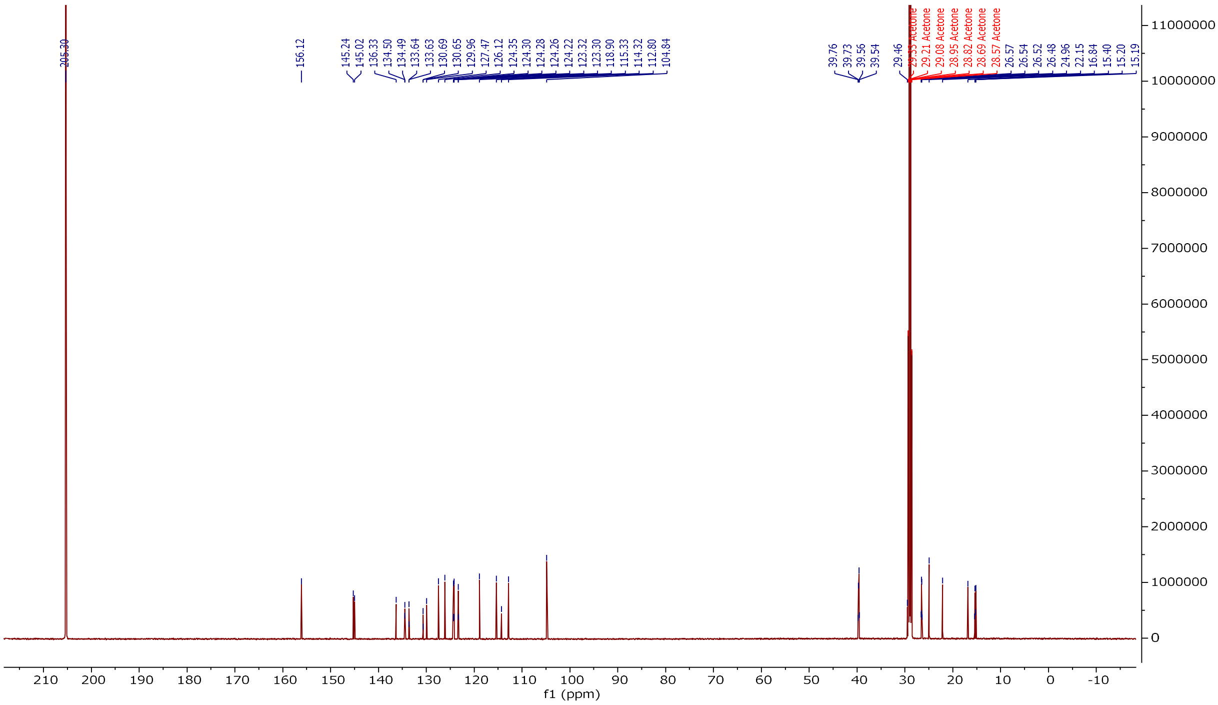

Figure S38: ^{13}C-NMR (1254 MHz, acetone –*d*6) spectrum of **9**

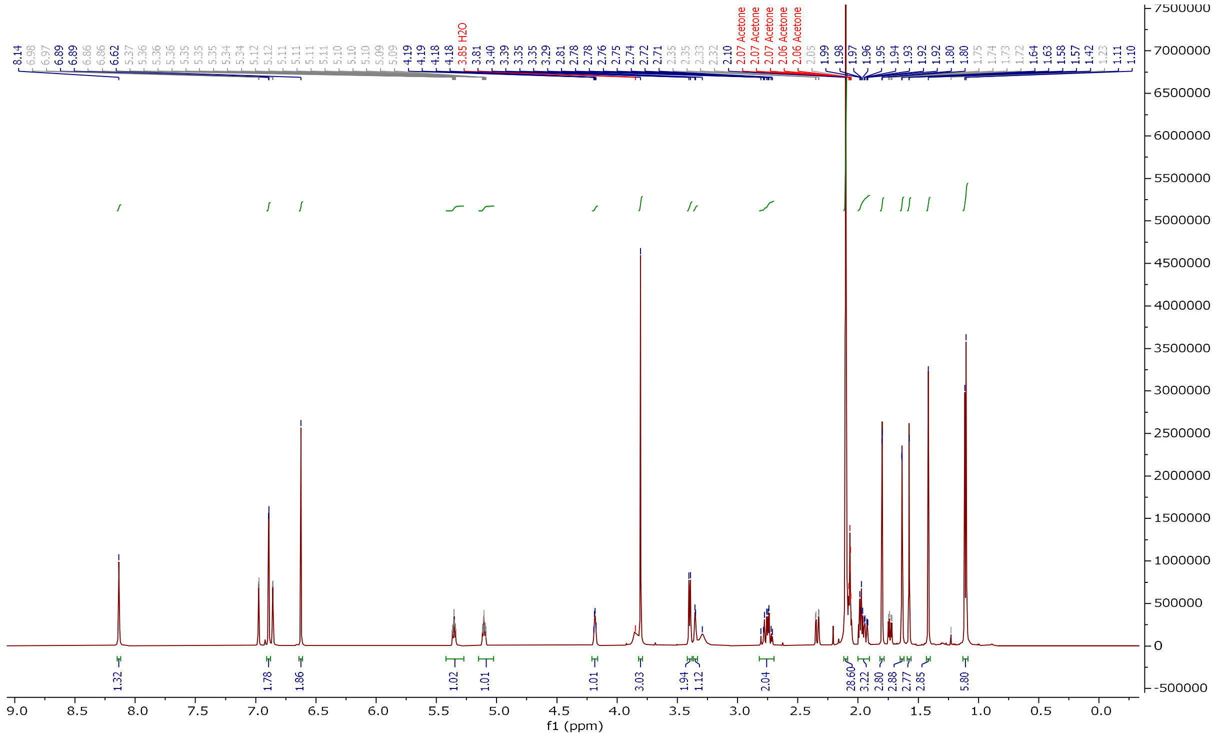

Figure S39: ^{1}H-NMR (500 MHz, acetone –d_6) spectrum of **10**

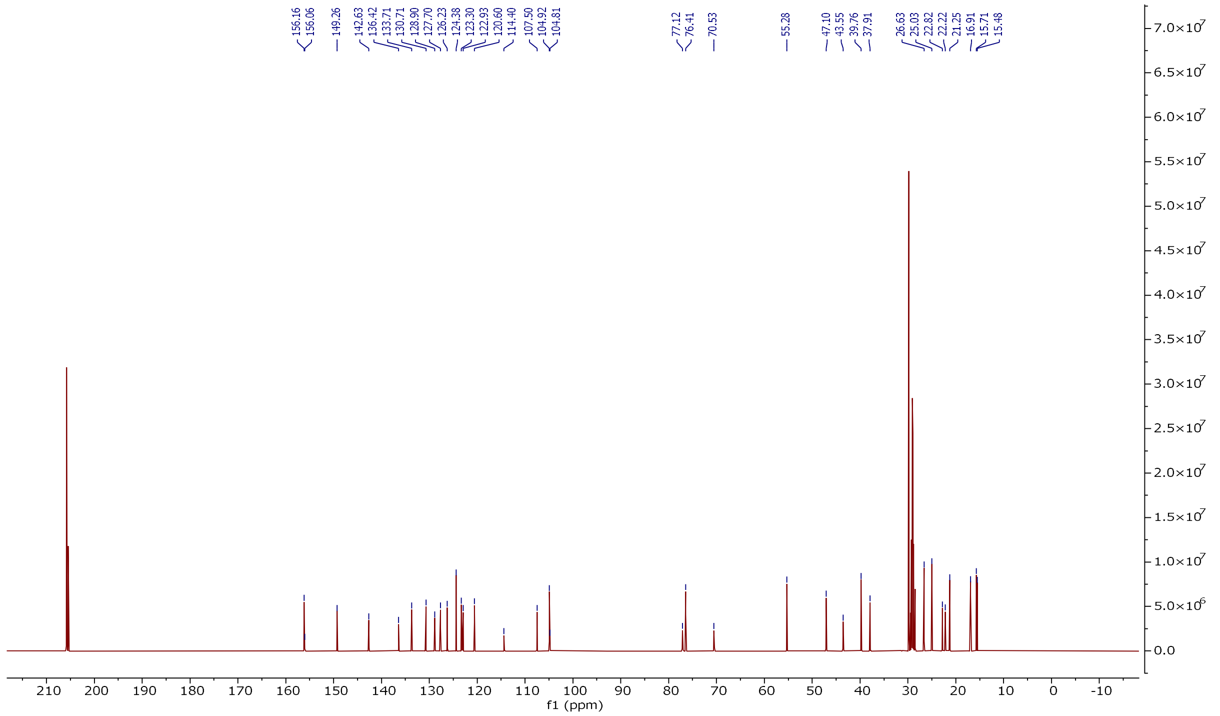

Figure S40: ^{13}C-NMR (1254 MHz, acetone –d_6) spectrum of **10**

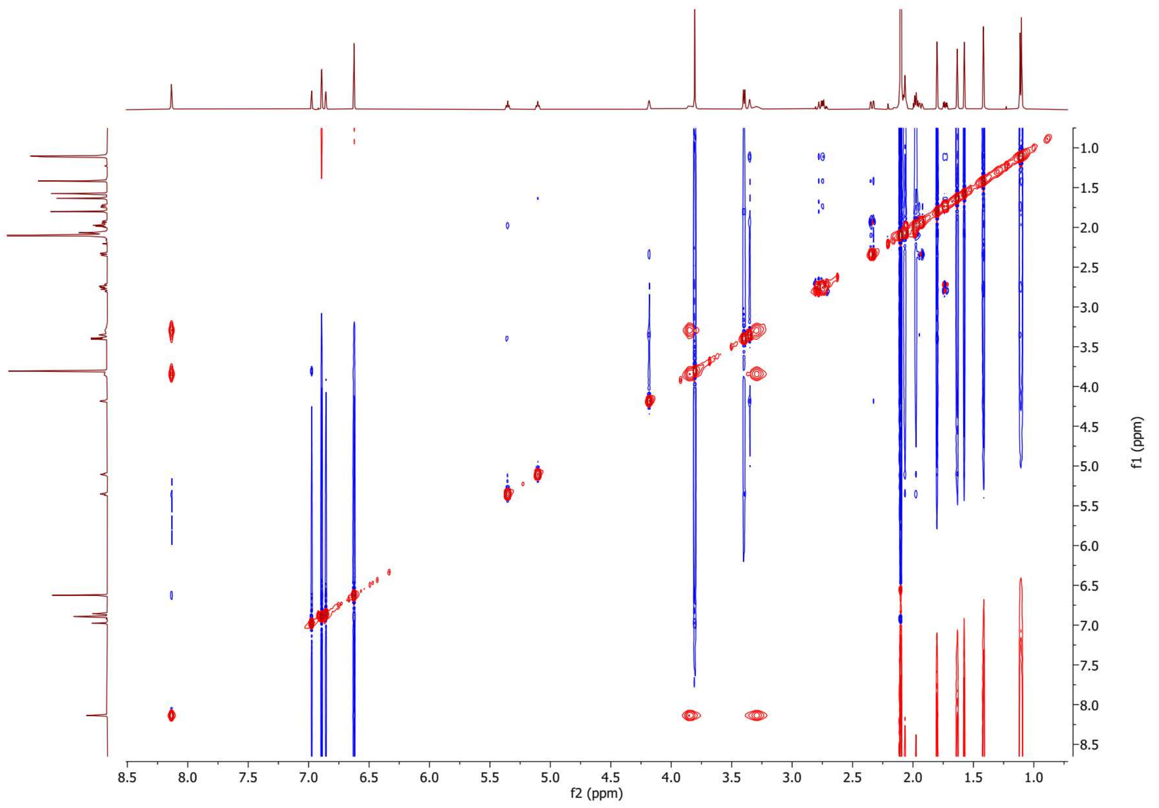

Figure S41: NOESY spectrum of **10**

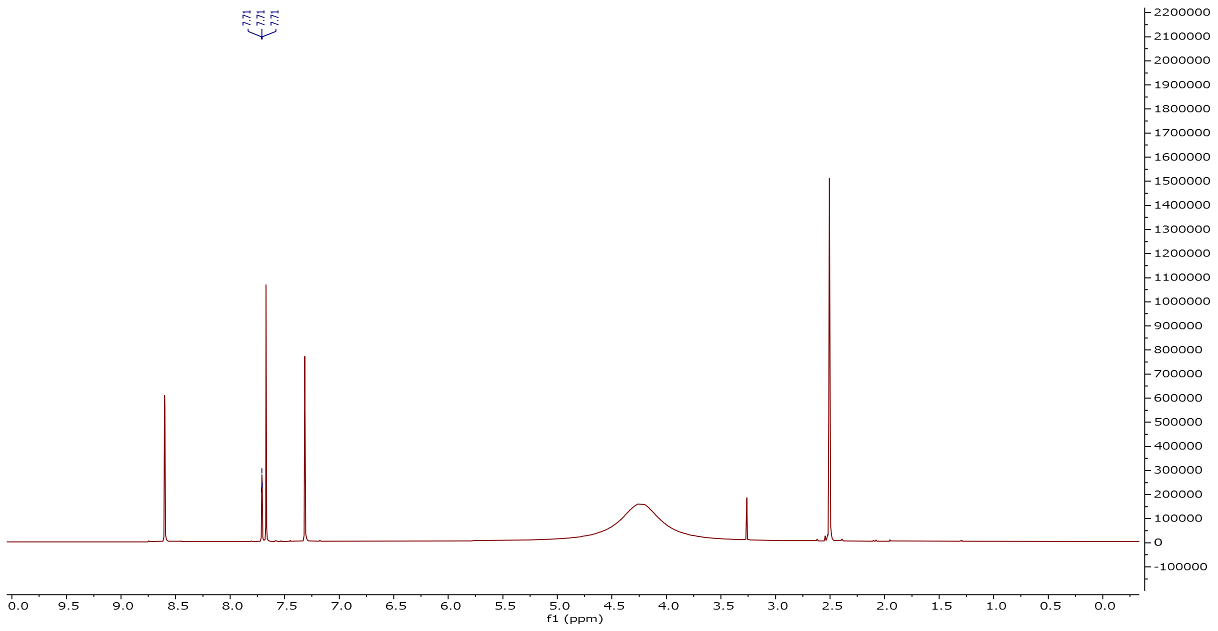

Figure S42: ^{1}H-NMR (500 MHz, Pyridine–d_5) spectrum of **11**

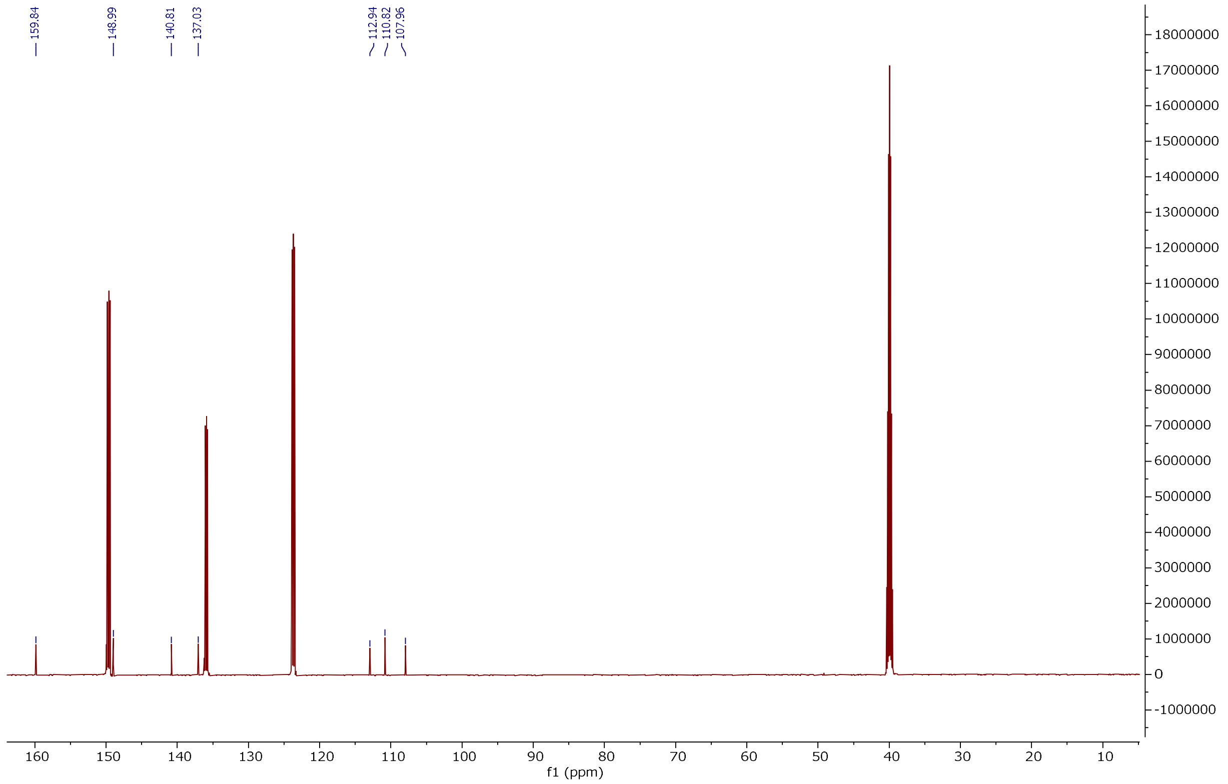

Figure S43: ^{13}C-NMR (125 MHz, Pyridine–d_5) spectrum of **11**

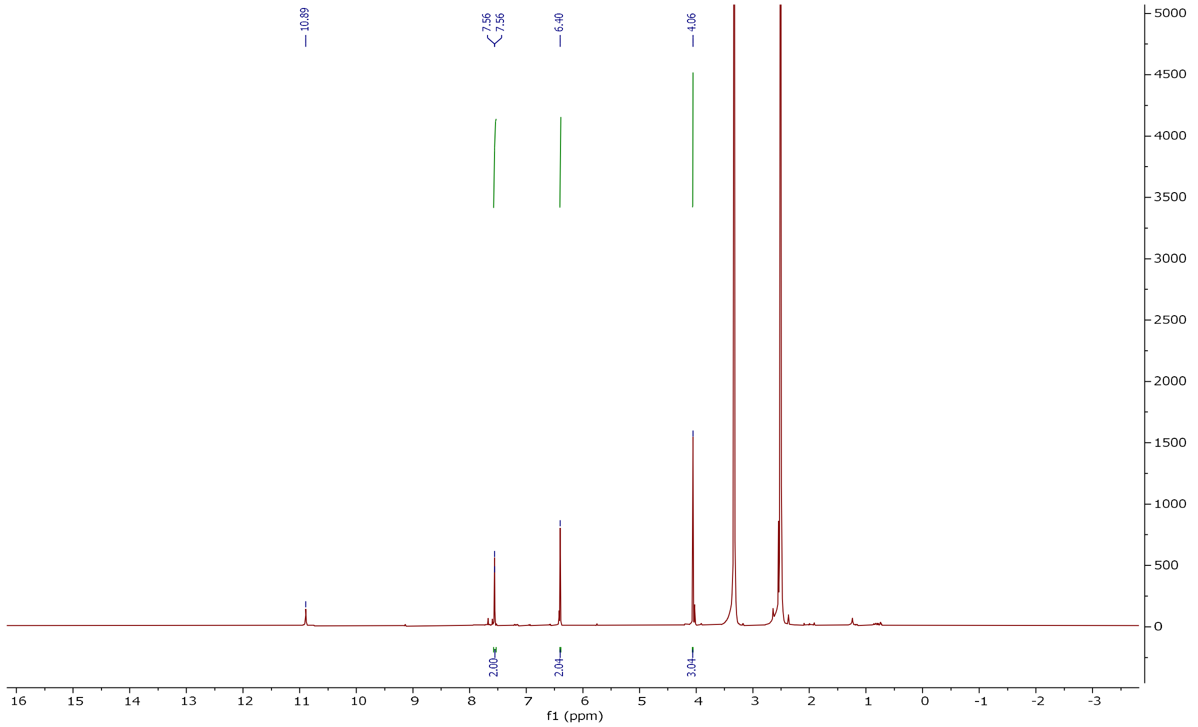

Figure S44: ^{1}H-NMR (500 MHz, DMSO–d_6) spectrum of **12**

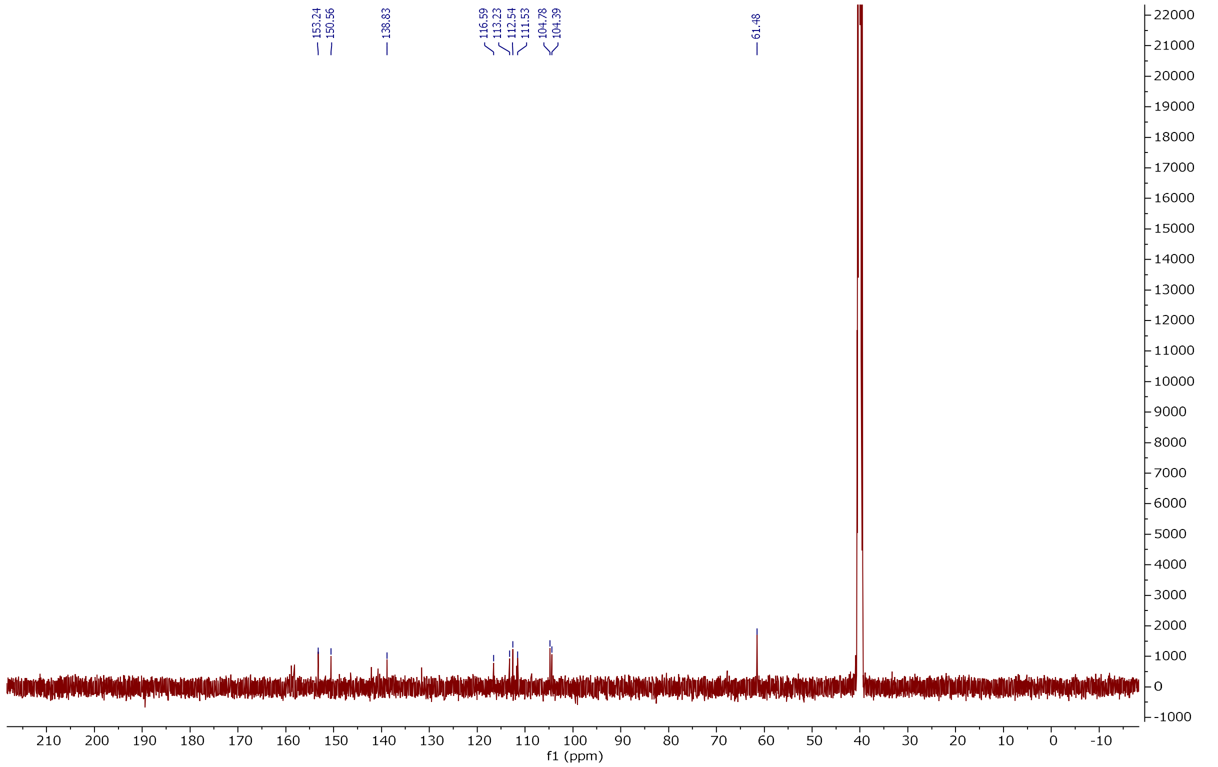

Figure S45: ^{13}C-NMR (125 MHz, DMSO–d_6) spectrum of **12**

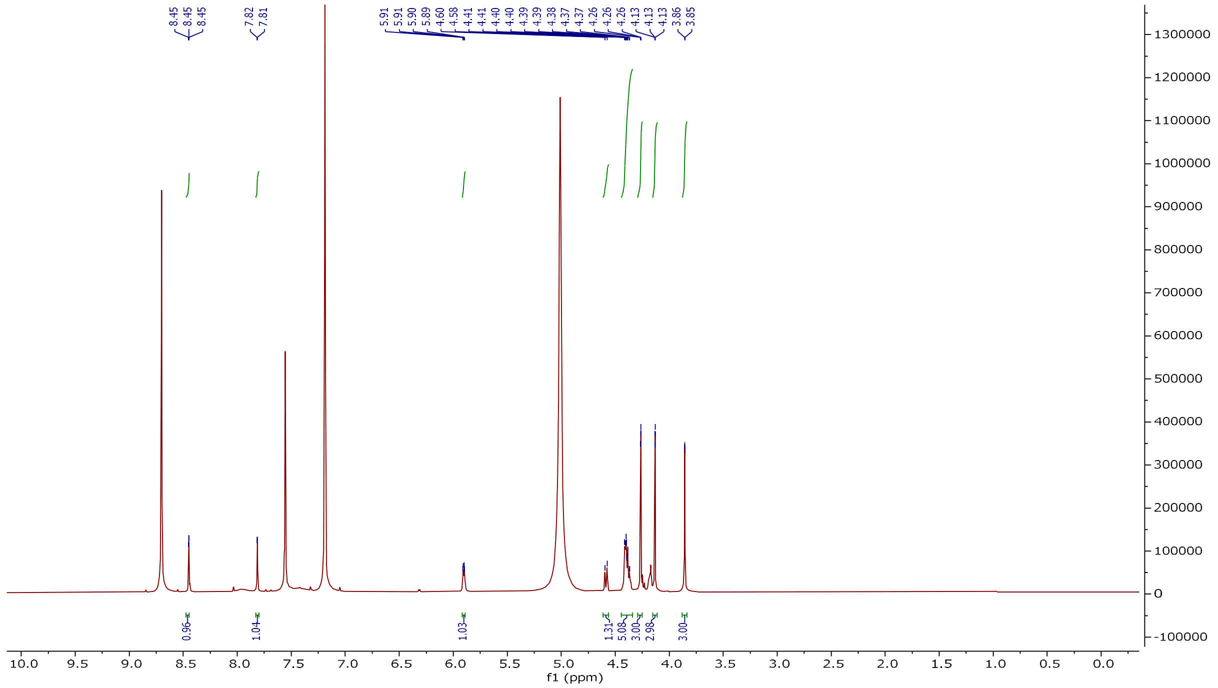

Figure S46: ^{1}H-NMR (500 MHz, Pyridine–d_5) spectrum of **13**

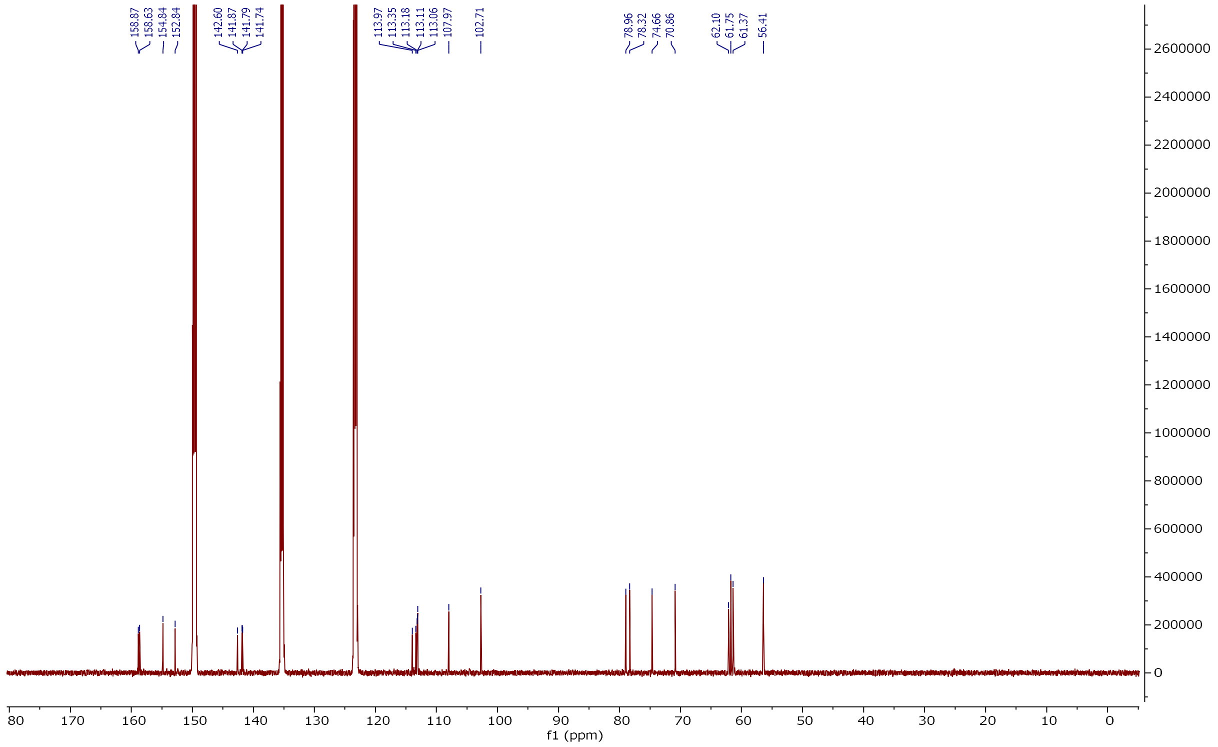

Figure S47: ^{13}C-NMR (125 MHz, Pyridine–d_5) spectrum of **13**

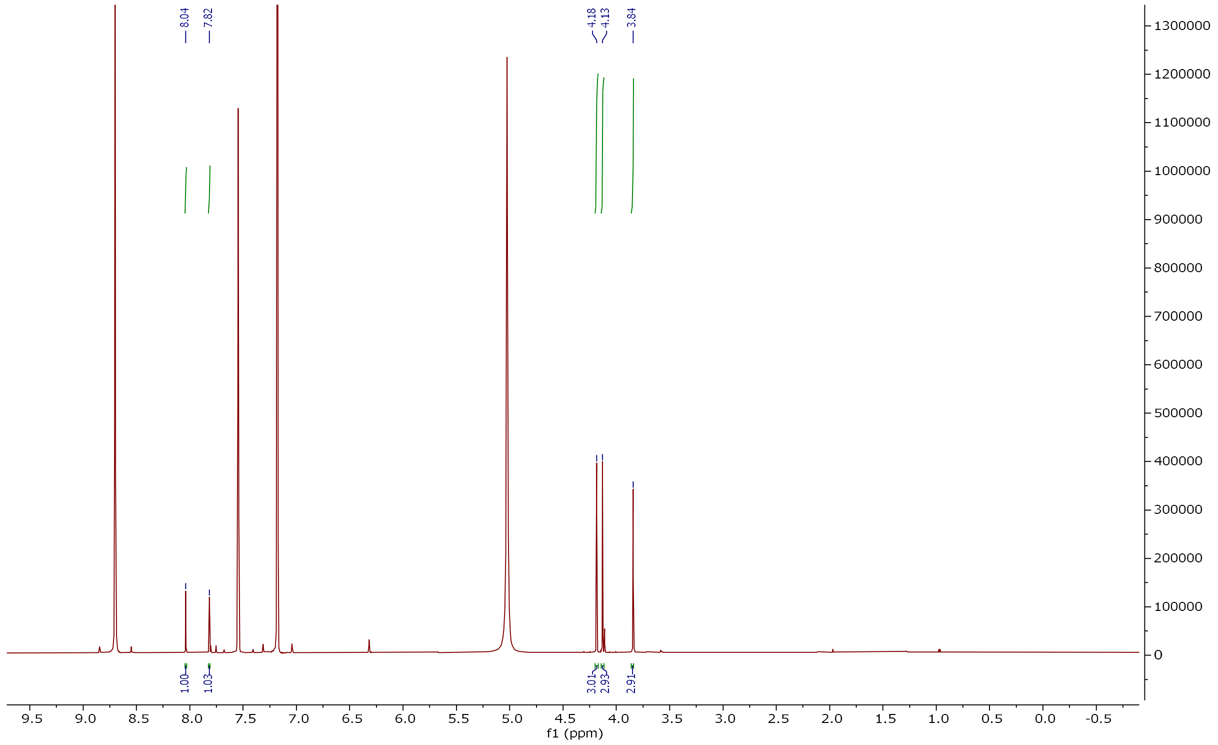

Figure S48: ^{1}H-NMR (500 MHz, Pyridine–d_5) spectrum of **14**

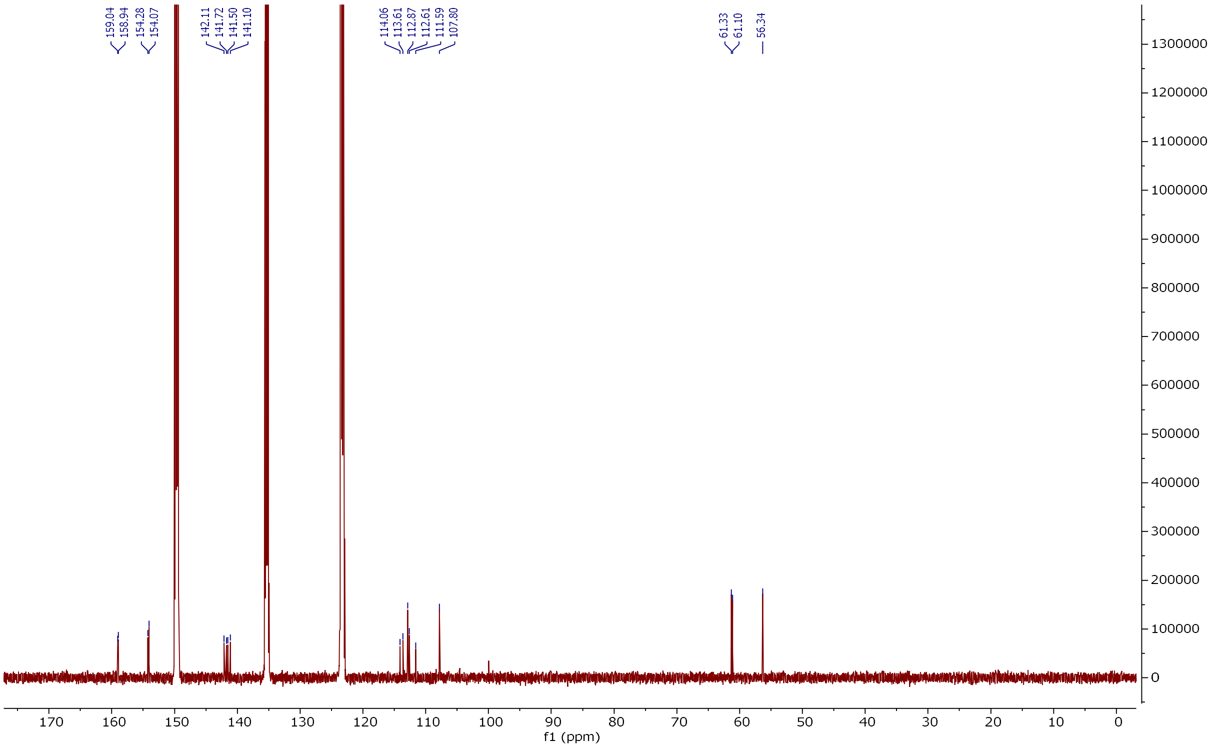

Figure S49: ^{13}C-NMR (125 MHz, Pyridine–d_5) spectrum of **14**

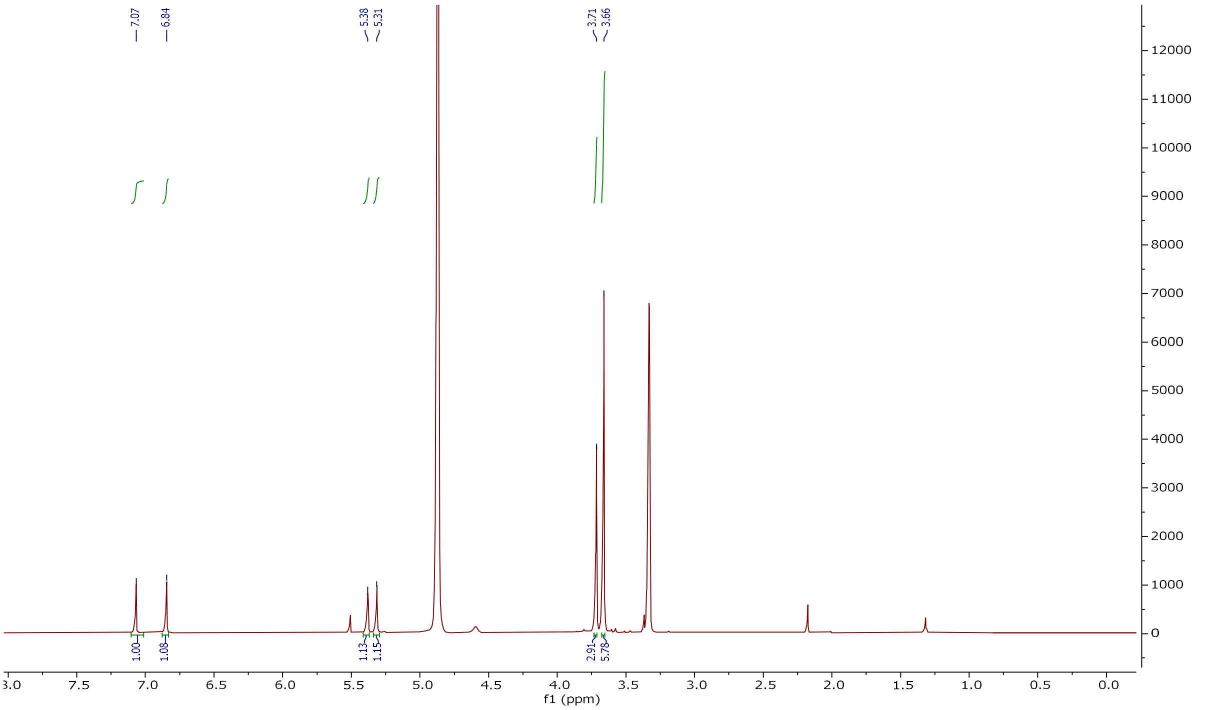

Figure S50: ^{1}H-NMR (500 MHz, CD_3OD) spectrum of **15**

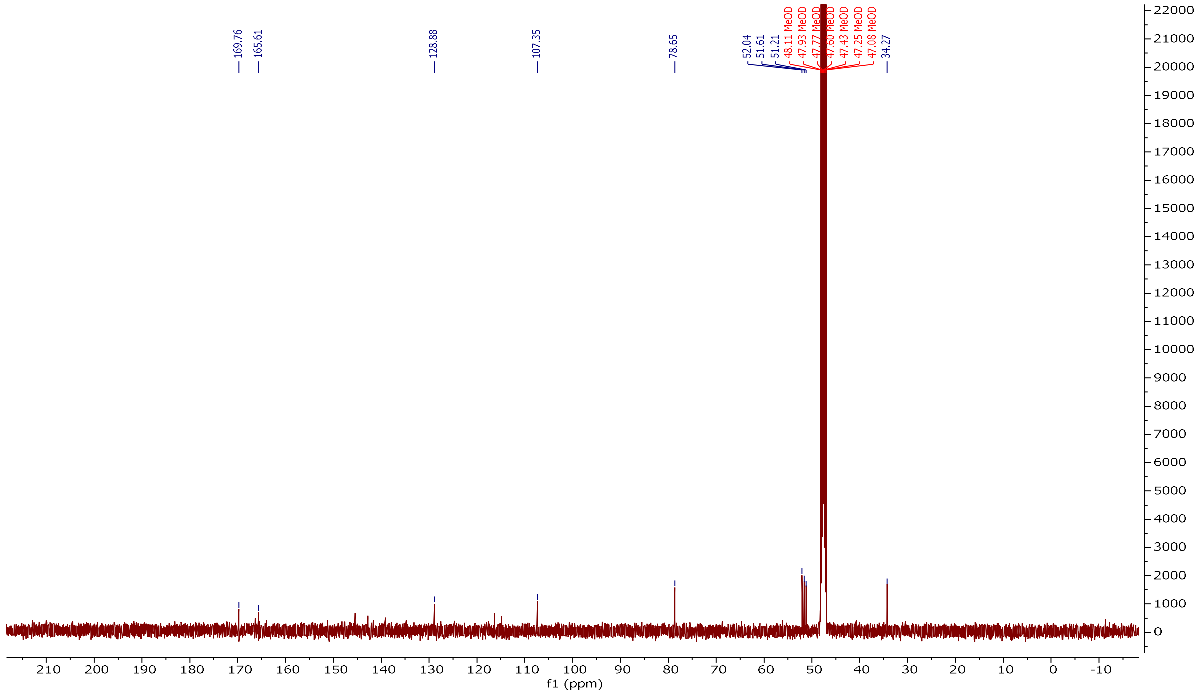

Figure S51: ^{13}C-NMR (125 MHz, CD_3OD) spectrum of **15**

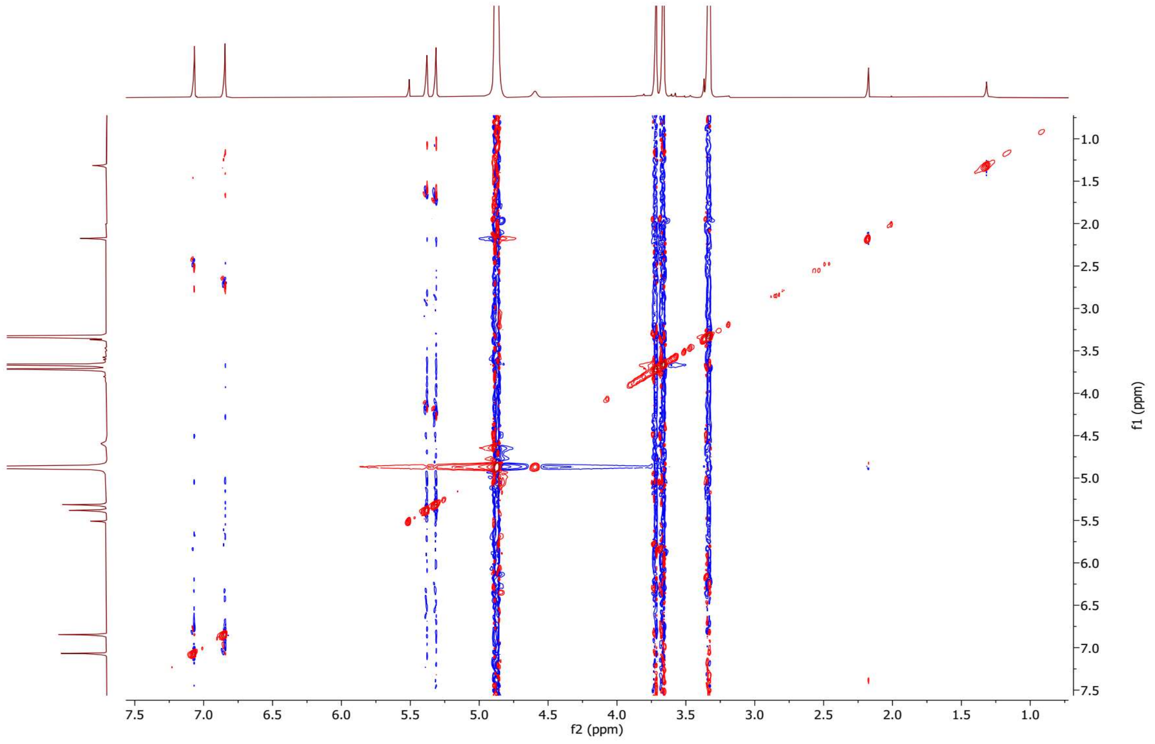

Figure S52: NOESY spectrum of **15**

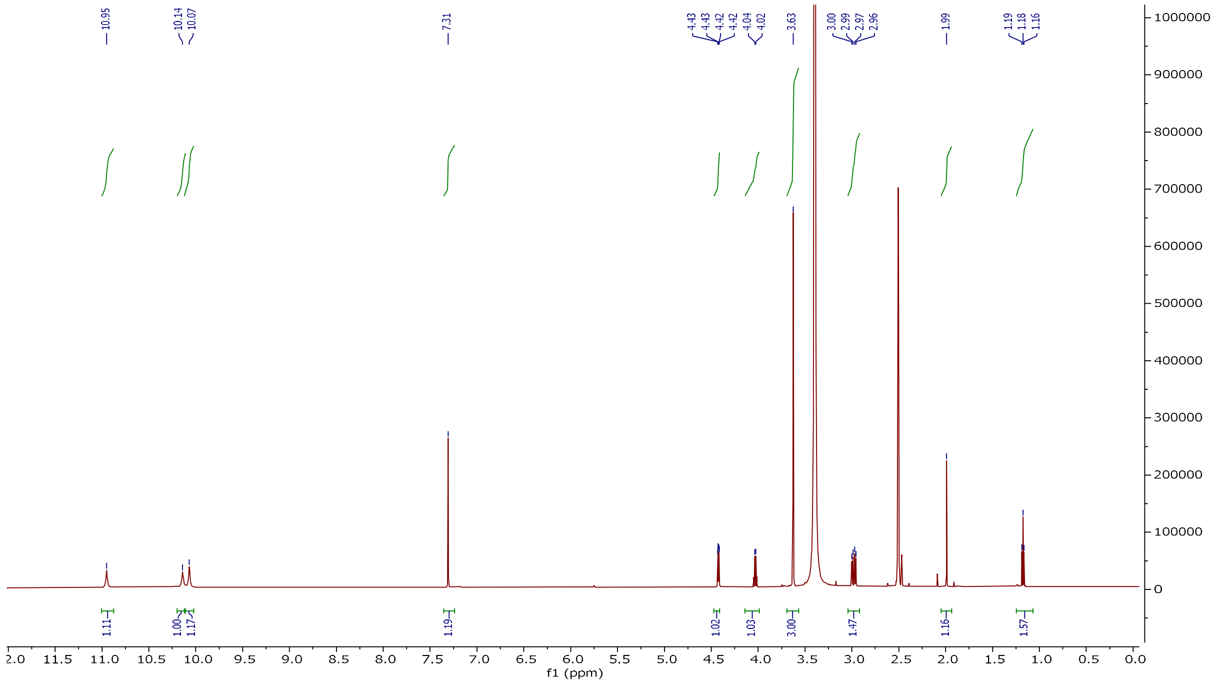

Figure S53: ^{1}H-NMR (500 MHz, DMSO–d_6) spectrum of **16**

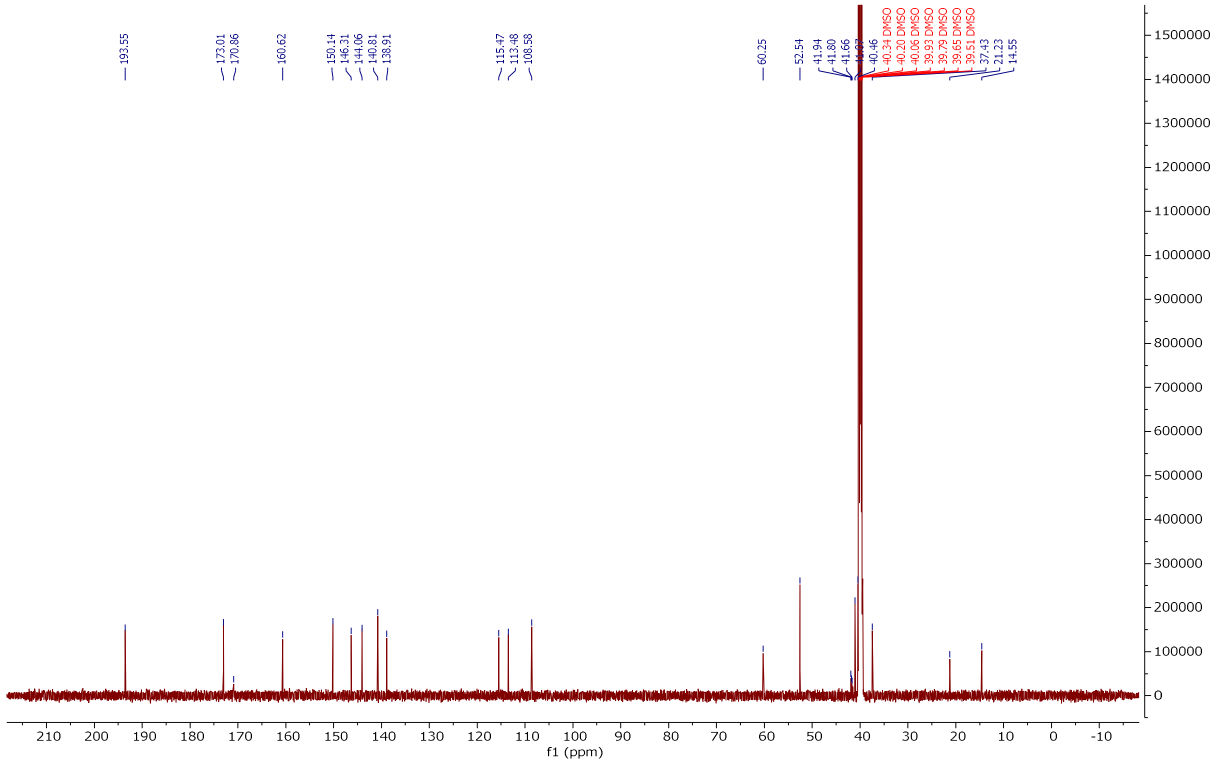

Figure S54: ^{13}C-NMR (125 MHz, *DMSO–d_6*) spectrum of **16**

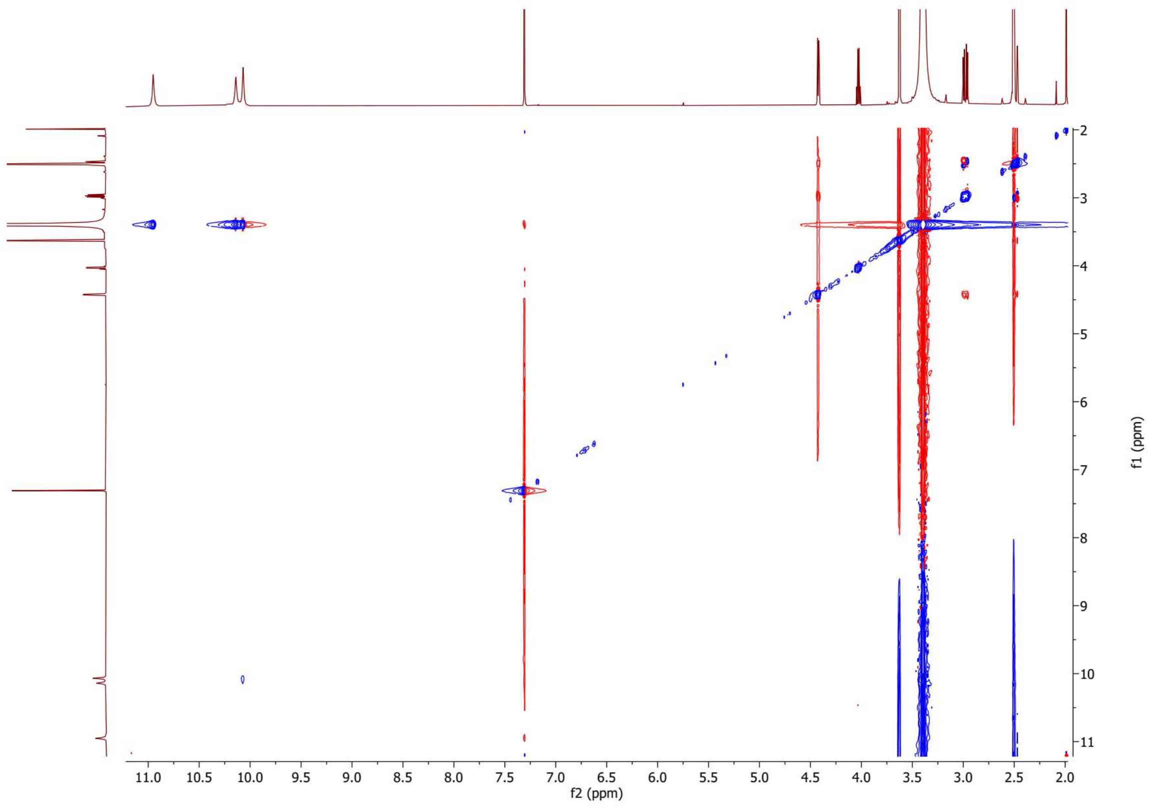

Figure S55: NOESY spectrum of **16**

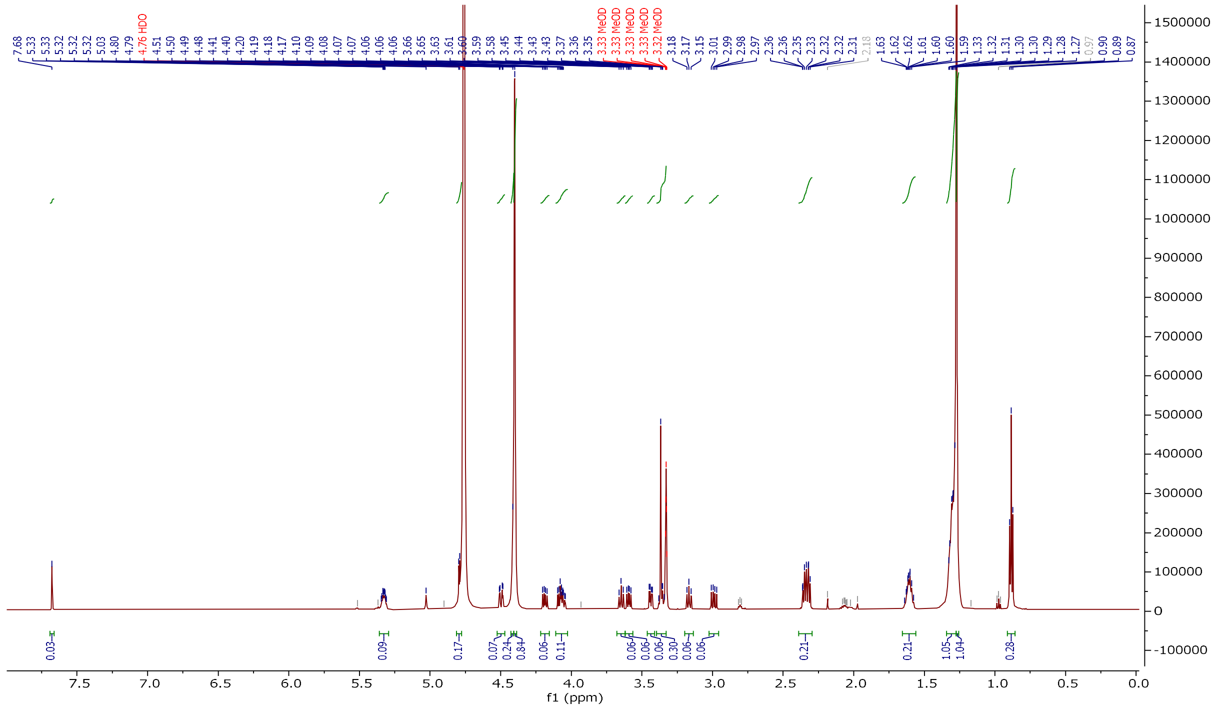

Figure S56: ^{1}H-NMR (500 MHz, $CDCl_3$-CD_3OD) spectrum of **17**

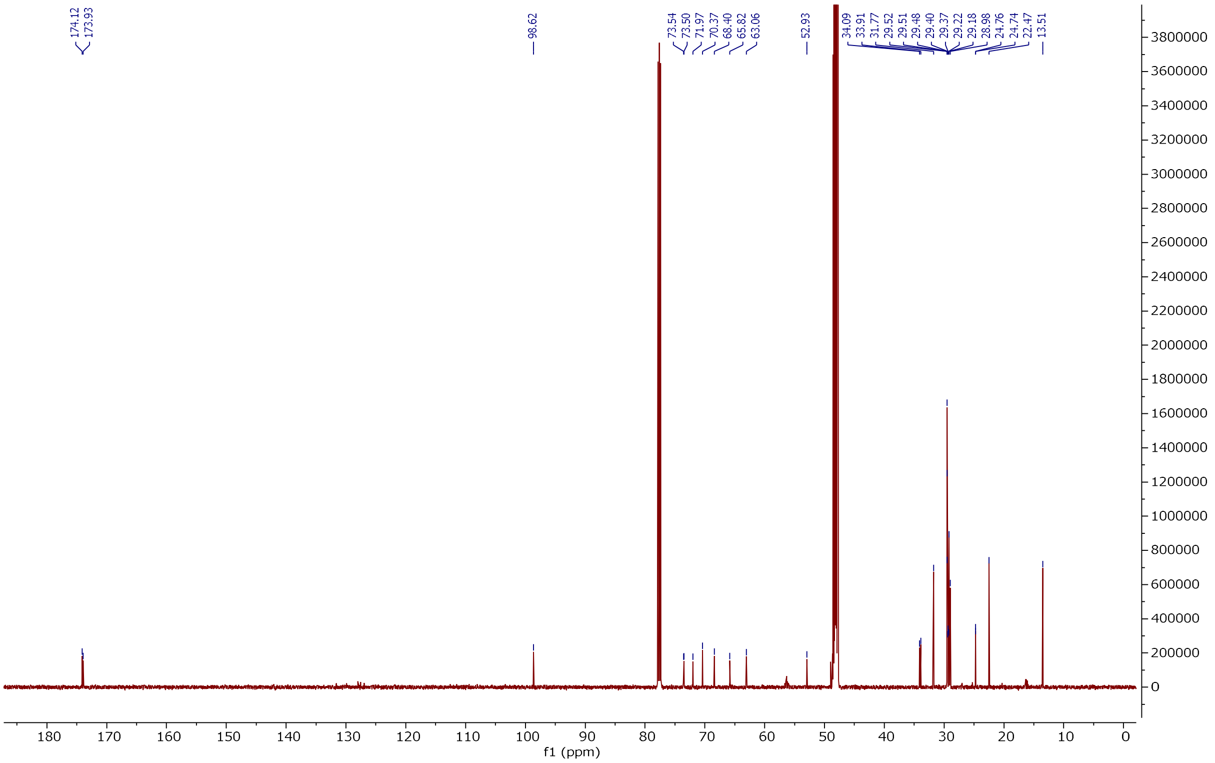

Figure S**57**: ^{13}C-NMR (125 MHz, $CDCl_3$-CD_3OD) spectrum of **17**

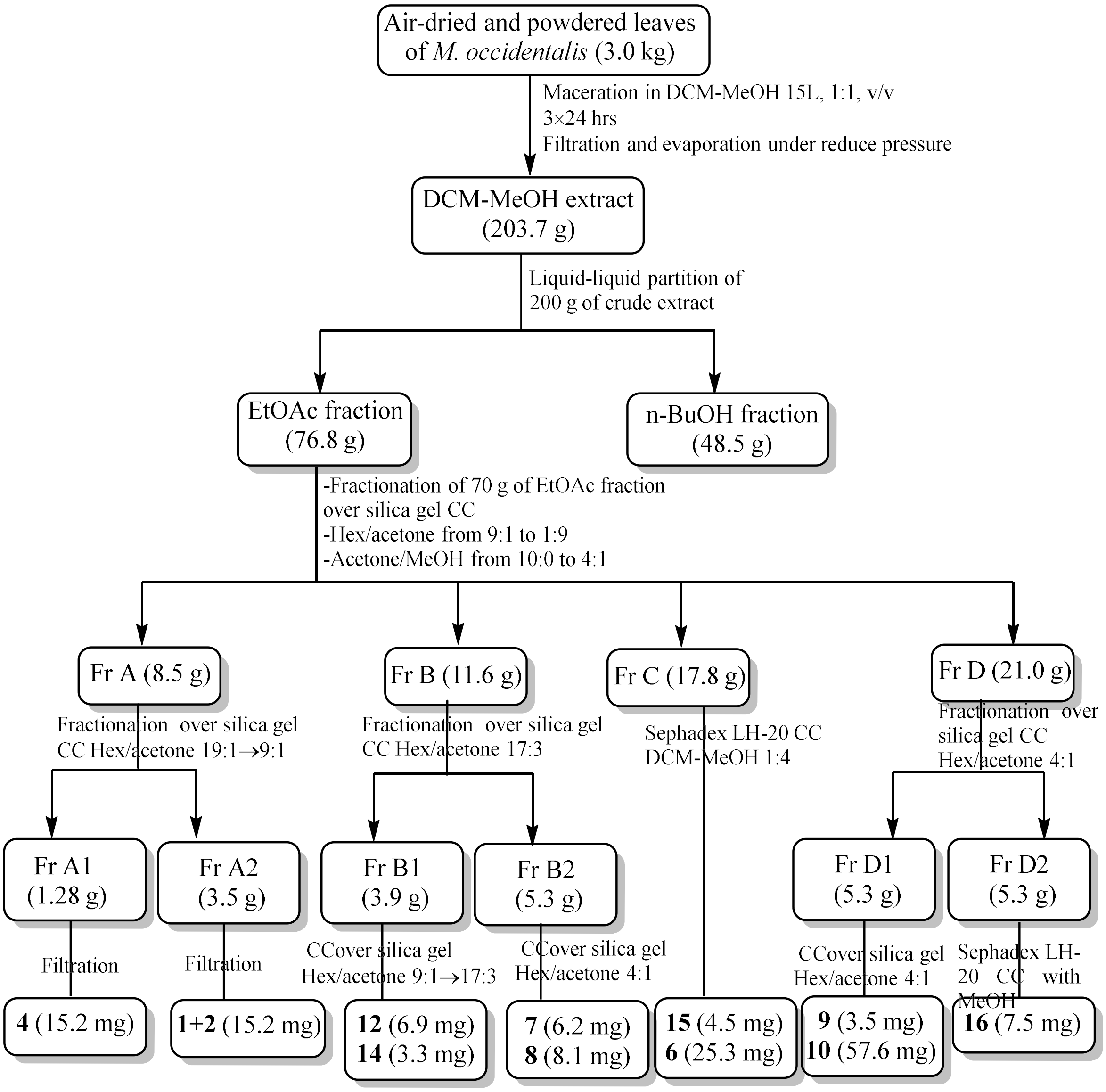

Air-dried and powdered leaves of *M. occidentalis* (3.0 kg)
Maceration in DCM-MeOH 15L, 1:1, v/v
3×24 hrs
Filtration and evaporation under reduce pressure
DCM-MeOH extract (203.7 g)
Liquid-liquid partition of 200 g of crude extract
EtOAc fraction (76.8 g)
n-BuOH fraction (48.5 g)
-Fractionation of 70 g of EtOAc fraction over silica gel CC
-Hex/acetone from 9:1 to 1:9
-Acetone/MeOH from 10:0 to 4:1
Fr A (8.5 g)
Fr B (11.6 g)
Fr C (17.8 g)
Fr D (21.0 g)
Fractionation over silica gel CC Hex/acetone 19:1→9:1
Fractionation over silica gel CC Hex/acetone 17:3
Sephadex LH-20 CC DCM-MeOH 1:4
Fractionation over silica gel CC Hex/acetone 4:1
Fr A1 (1.28 g)
Fr A2 (3.5 g)
Fr B1 (3.9 g)
Fr B2 (5.3 g)
Fr D1 (5.3 g)
Fr D2 (5.3 g)
Filtration
Filtration
CCover silica gel Hex/acetone 9:1→17:3
CCover silica gel Hex/acetone 4:1
CCover silica gel Hex/acetone 4:1
Sephadex LH-20 CC with MeOH
4 (15.2 mg)
1+2 (15.2 mg)
12 (6.9 mg)
14 (3.3 mg)
7 (6.2 mg)
8 (8.1 mg)
15 (4.5 mg)
6 (25.3 mg)
9 (3.5 mg)
10 (57.6 mg)
16 (7.5 mg)

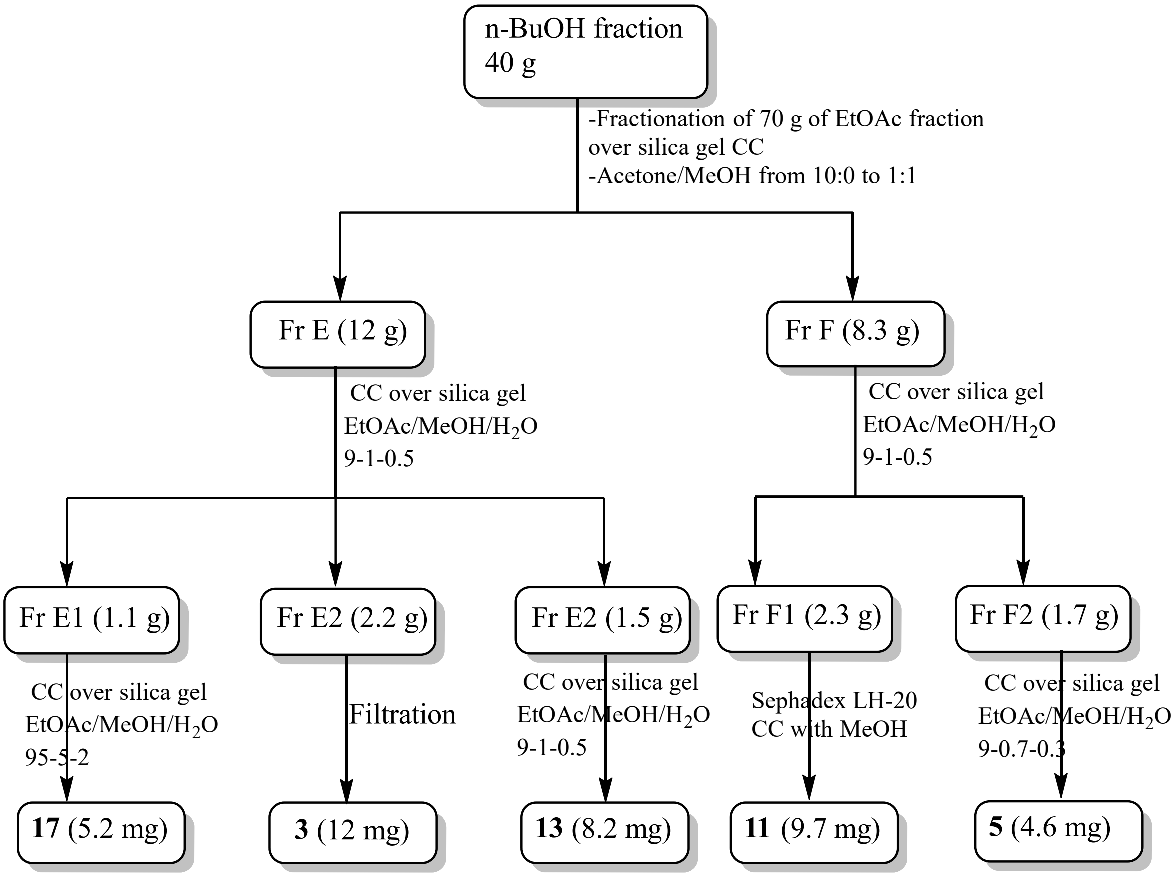

Scheme S1. Protocole of extraction and isolation of compounds from *M. occidentalis*
